# Cambrian euarthropod *Urokodia aequalis* sheds light on the origin of Artiopoda body plan

**DOI:** 10.1016/j.isci.2024.110443

**Published:** 2024-07-11

**Authors:** Cong Liu, Dongjing Fu, Yu Wu, Xingliang Zhang

**Affiliations:** 1 State Key Laboratory of the Continental Dynamics, Shaanxi Key Laboratory of Early Life and Environments, Department of Geology, Northwest University, Xi’an 710069, China; 2 Nanjing Institute of Geology and Paleontology, Chinese Academy of Sciences, Nanjing 210008, China

**Keywords:** Zoology, Evolutionary biology, Systematics

## Abstract

The origin and evolution of trilobated body plan of the Artiopoda, a group of epibenthic euarthropods from Cambrian Lagerstätten, remain unclear. Here we examine old and new specimens of *Urokodia aequalis*, one of euarthropods from the Chengjiang biota, revealing new morphological details and revising its taxonomy. *Urokodia* possesses an elongate body with a five-segmented head, a thorax with 13–15 tergites, and a three-segmented pygidium with well-defined axial region. The ventral morphology includes paired stalked eyes, one fleshy antenna pair, the following homogeneous head and thoracic appendages, each with an annular proximal-element, an articulated stenopodous branch and a lamellar flap, and the pygidial appendages solely consisting of lamellar flaps. Cladistic analyses resolved *Urokodia* as the basal-most member of the Artiopoda, offering a hypothesis of the initial origin of trilobation in the pygidium. The new data, in conjunction with the presence of the elongated body plan across major lineages of euarthropods, suggest a convergent evolution of this trait.

## Highlights

• *Urokodia* is the earliest diverging taxon of the Artiopoda with an elongated body plan
• Its narrow head-thorax followed by a flat pygidium with a well-defined axial region
• *Urokodia* set a hypothesis that the initial origin of trilobation in the pygidium

## Graphical abstract

**Figure:**
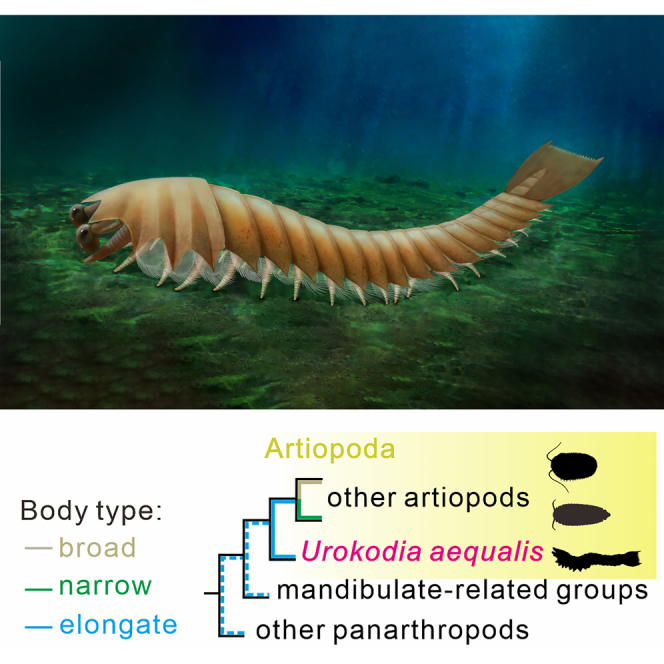

## Introduction

The Artiopoda represents a crucial clade of euarthropods, displaying immense diversity and abundance across Cambrian Lagerstätten.^1^^,^^2^^,^^3^^,^^4^^,^^5^ Members of this clade, including trilobites and their lightly skeletonized close relatives, such as nektaspids, concilitergans, and vicissicaudatans, are generally characterized by broad dorsal exoskeleton.^3^^,^^6^^,^^7^ Despite the examination of animal genomes and in-depth analyses of exceptionally preserved Cambrian fossils allowing a deep understanding on the origin and the evolution of arthropodan body plan,^8^^,^^9^ the origin and the evolution of the specialized body plan of artiopods remain insufficiently understood.

*Urokodia aequalis*, originally described by Hou, Chen, and Lu, 1989, is a rare euarthropod characterized by its distinctive elongate body. Reported from the 518 million-year-old Chengjiang biota, this species was initially identified as a member of the Mollisoniida (stem group of chelicerates) solely based on the morphology of its dorsal exoskeleton.^3^^,^^10^^,^^11^^,^^12^ However, our observations of new and published specimens using optical and micro-computed tomography (micro-CT) techniques help unveiling its ventral morphology (Videos S1 and S2), thus allowing a more comprehensive understanding of its overall morphology. Phylogenic analyses have since resolved *U. aequalis* as the basal member of the Artiopoda, providing a new elongated body plan among this lineage and allowing for the estimation of artiopodan body evolution.

**Figure 1:**
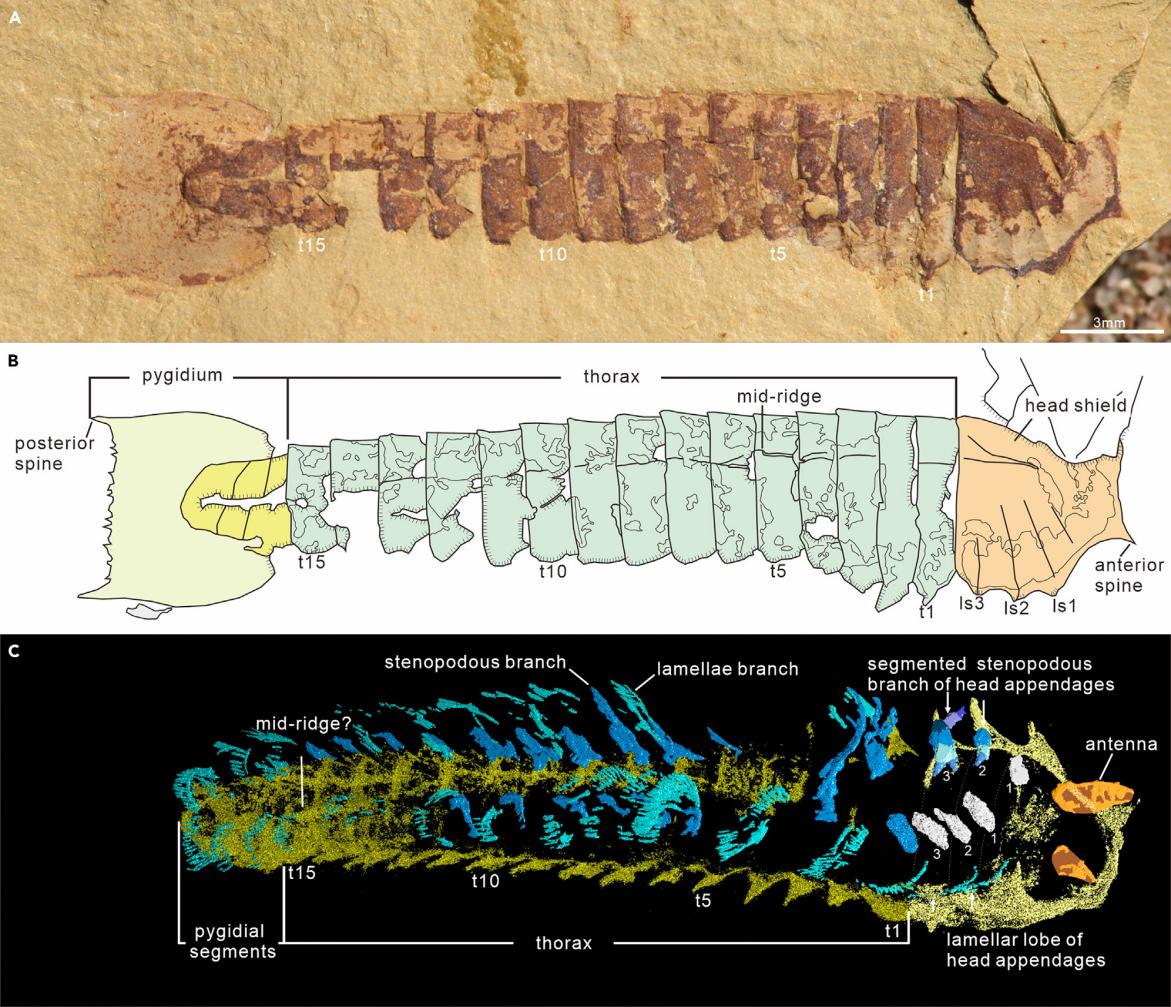
General morpho-anatomy of *Urokodia aequalis* from the Chengjiang biota (A) SJZ-B16-078, a complete, twisted specimen, anterior part in oblique view, posterior region (pygidial shield) in dorsal view; showing dissimilarity between head and pygidial shield. (B) Camera-lucida drawing of (A), showing the composition of the dorsal exoskeleton. (C) Rendering micro-CT data in ventral side, showing a pair of antennae and three pairs of post-antennal head appendages, a series of thoracic appendages, and three pairs of lamellar lobes of pygidial appendages. ls, lateral spines of head shield; t, thoracic segment; numbers refer to the topological position of the corresponding structure with reference to the description.

Video S1. Raw scanning data of Micro-CT of SJZ-B16-078, related to Figure 1

Video S2. Raw scanning data of Micro-CT of SJZ-B21-078, related to Figure 3

## Results

### Systematic paleontology

Phylum Euarthropoda Lankester, 1904.

Artiopoda Hou and Bergström, 1997.

Genus *Urokodia* Hou, Chen, and Lu, 1989.

Type species. *Urokodia aequalis* Hou, Chen & Lu, 1989.

Diagnosis (emended from Zhang et al.^12^). Artiopod with an elongate exoskeleton, composed of a head shield, up to 15 articulated thoracic tergites, and a flattened pygidial shield. Head shield sub-rectangular in dorsal view, vaulted dorsally, carrying a pair of anterior spines followed by three pairs of lateral spines, each corresponding to a transverse ridge on the lateral region of the head shield, and covering a pair of stalked eyes, a pair of antennae and three pairs of post-antennal appendages. Thorax vaulted dorsally, no axial region, and composed of up to 15 tergites, with each covering a pair of appendages. Pygidial shield trapezoid in outline, bearing a pair of posterior spines and numerous small spines on the straight posterior margin, axial region well-defined, composed of three segments, each carrying a pair of appendages.

Remarks. Twenty-one specimens of *Urokodia* were previously described,^3^^,^^10^^,^^11^^,^^12^ primarily focusing on the dorsal morphology of the exoskeleton. In this study, additional 29 specimens are analyzed, providing a considerable amount of new morphological information, revealing previously undocumented features such as the transverse impressions on the head shield and variations in the number of thoracic tergites, ranging from 13 to 15. Importantly, examination of ventral soft tissues shed further light on the limb structures of *Urokodia*. Consequently, *Urokodia* is assigned to the Artiopoda on the basis of consensus phylogenetic results using Bayesian inference and maximum parsimony methods.

*Urokodia aequalis*; Hou, Chen, and Lu, 1989 (figs. 3 and 4).

Holotype. NIGPAS 108311(Hou et al.,^10^ Pl. Ⅴ, fig. 1), from the Maotianshan, level M_2_.

Occurrence. Specimens were sampled from six localities of the Chengjiang biota, i.e., the Erjie, Jianshan, Haoyicun, Mafang, Sanjiezi, and Shankou (EJ, JS, HY, MF, SJZ, and SK, respectively), Yu’anshan shale Member (or Yu’anshan Formation) of the Helinpu Formation (Chiungchussu Formation), *Eoredlichia*-*Wutingaspis* zone, Cambrian Series 2, Stage 3, Yunnan Province, China.

**Figure 2:**
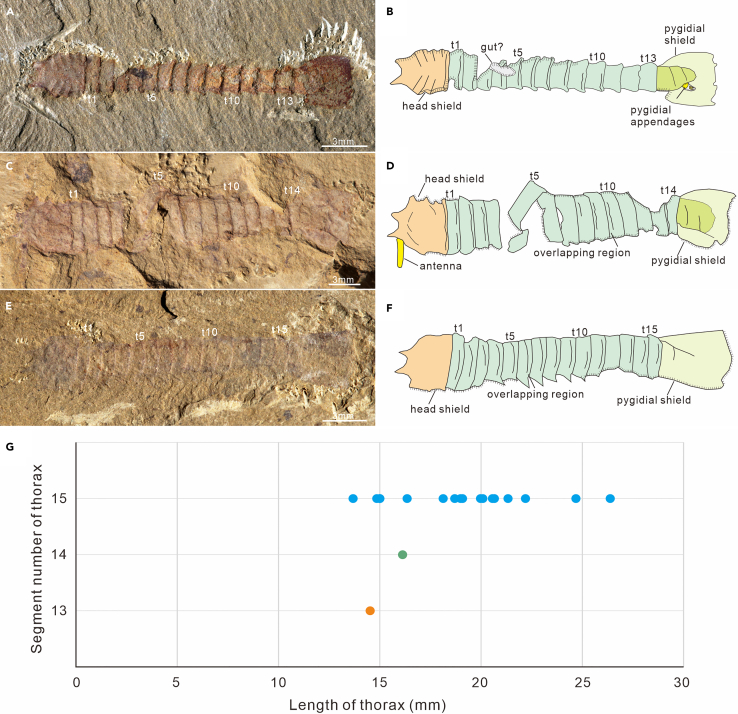
Phenotypes of *Urokodia* thorax and the statistical measurements of body length (A and B) SK-1573, dorsal view, showing 13 thoracic tergites and a lamellar lobe of pygidium. (C and D) MF-1581B, dorsal view, showing 14 thoracic tergites and a linear antenna. (E and F) JS-1546, dorsal view, showing the smallest specimen with 15 thoracic tergites. (G) The statistical measurements showing variations of the segment number and the total length of the thorax. Note the exception that the smallest specimen bears 15 thoracic tergites, and its thorax is shorter than that of the 14-tergite phenotype.

Preservation. Specimens occur in a variety of orientations respecting to the bedding. Nineteen intact individuals have been used to analyze preservation styles, including 15 oblique specimens that demonstrate three types of orientation—two twisted specimens with the anterior body laterally flattened while the posterior body in dorsal view, e.g., SJZ-B16-078 in Figure 1; four in lateral vision but showing a slight dorsal deflection, e.g., EJ-1561A in Figure S1; nine in lateral view, e.g., EJ-1560A in Figure 2E); and four parallel specimens in dorsal view, e.g., SK-1573 in Figure 2A.

Description. The examination of 29 new *Urokodia* specimens has led to the identification of unrecognized traits in previous studies, including transverse impressions on the head shield, a pair of stalked eyes, and morphologies of the appendages. Additionally, new observations have prompted a reassessment of exoskeletal features, such as body flatness, marginal spines, and ridges on head shield, as well as the shape and alterations in the number of thoracic tergites, and the morphology of the pygidial shield. These findings are elaborated upon in the following description.

*Dorsal exoskeletons.* Elongate exoskeleton, ranging from 21.80 to 31.10 mm in length, and from 1.90 to 5.20 mm in width, divided into three regions: a head shield, thorax with 13–15 tergites, and a pygidial shield, accounting for 15%, 69%, and 16% of the total length, respectively. The exoskeleton was succinctly described, with the head shield depicted as an identical object to the pygidial shield (Hou et al.^10^). A subsequent report of a complete specimen demonstrated the critical differences between head and pygidial shields (Zhang et al.^12^). Yet, this interpretation was frequently questioned (Hou et al.^3^^,^^11^). New observations of old and new material refine the details of the exoskeleton and reconfirm the dissimilarity of head and pygidial shields.

**Figure 3:**
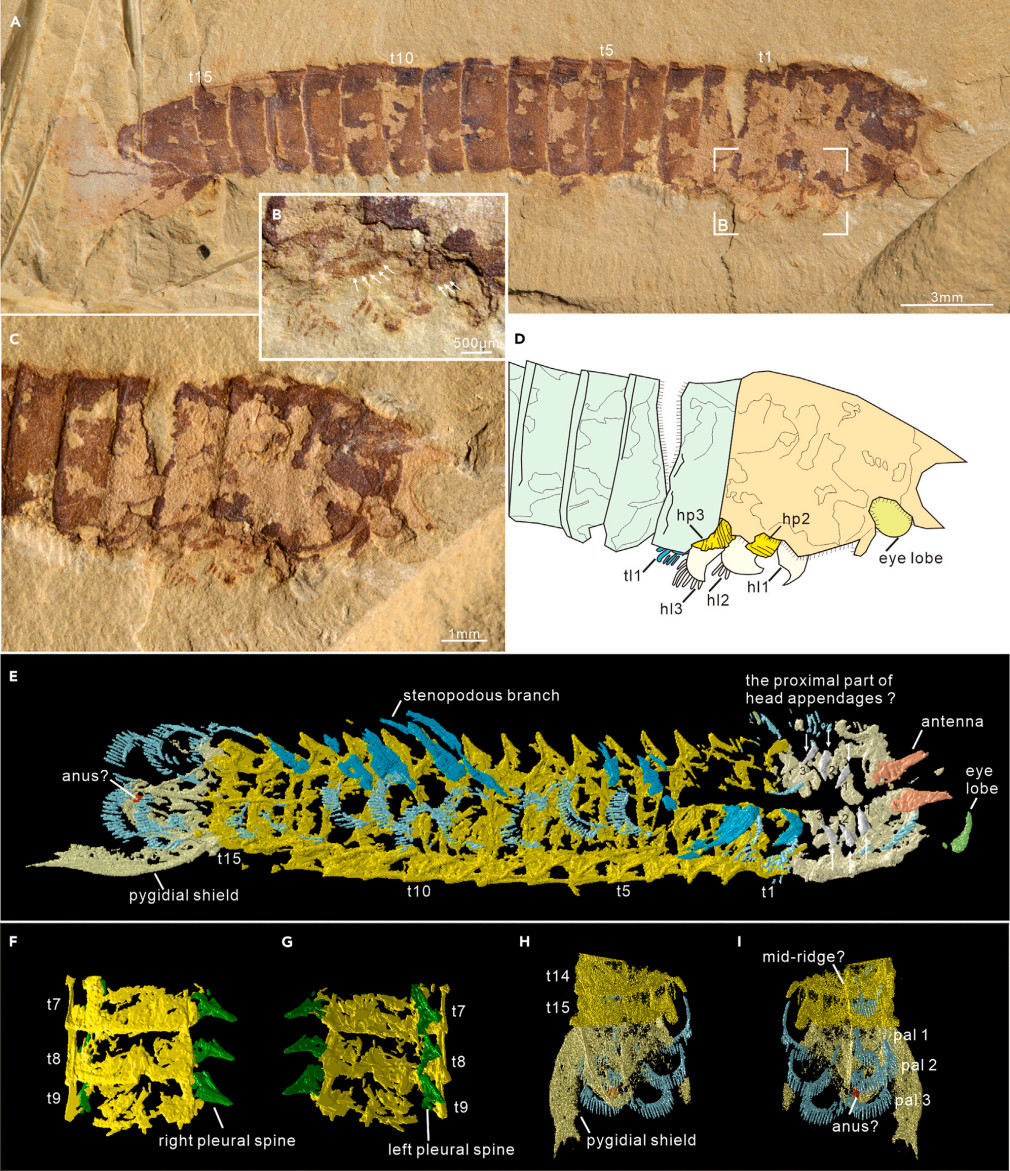
General morpho-anatomy and details of head appendicular structures of *Urokodia*, SJZ-B21-078 (A) General view of the slightly twisted specimen. (C and D) Close-up of head and anterior trunk region, showing one eye lobe, three lamellar lobes and two annulated branches (detailed in B). (E) Rendering micro-CT data in ventral side, showing an eye lobe, a pair of antennae, three pairs of post-antennal head appendages, a discontinuous series of thoracic appendages, and three pairs of flap-shaped pygidial appendages. (F and G) Details of the 7th to 9th thoracic tergites with pleural spines pointing ventrally. (H and I) Close-up of the pygidium, showing three pairs of lamellar lobes. (E)–(I) share the same scale as (A). hp, the proximal part of head appendages; hl, lamellar lobe of head appendages; pal, lamellar lobes of pygidial appendages.

**Figure 4:**
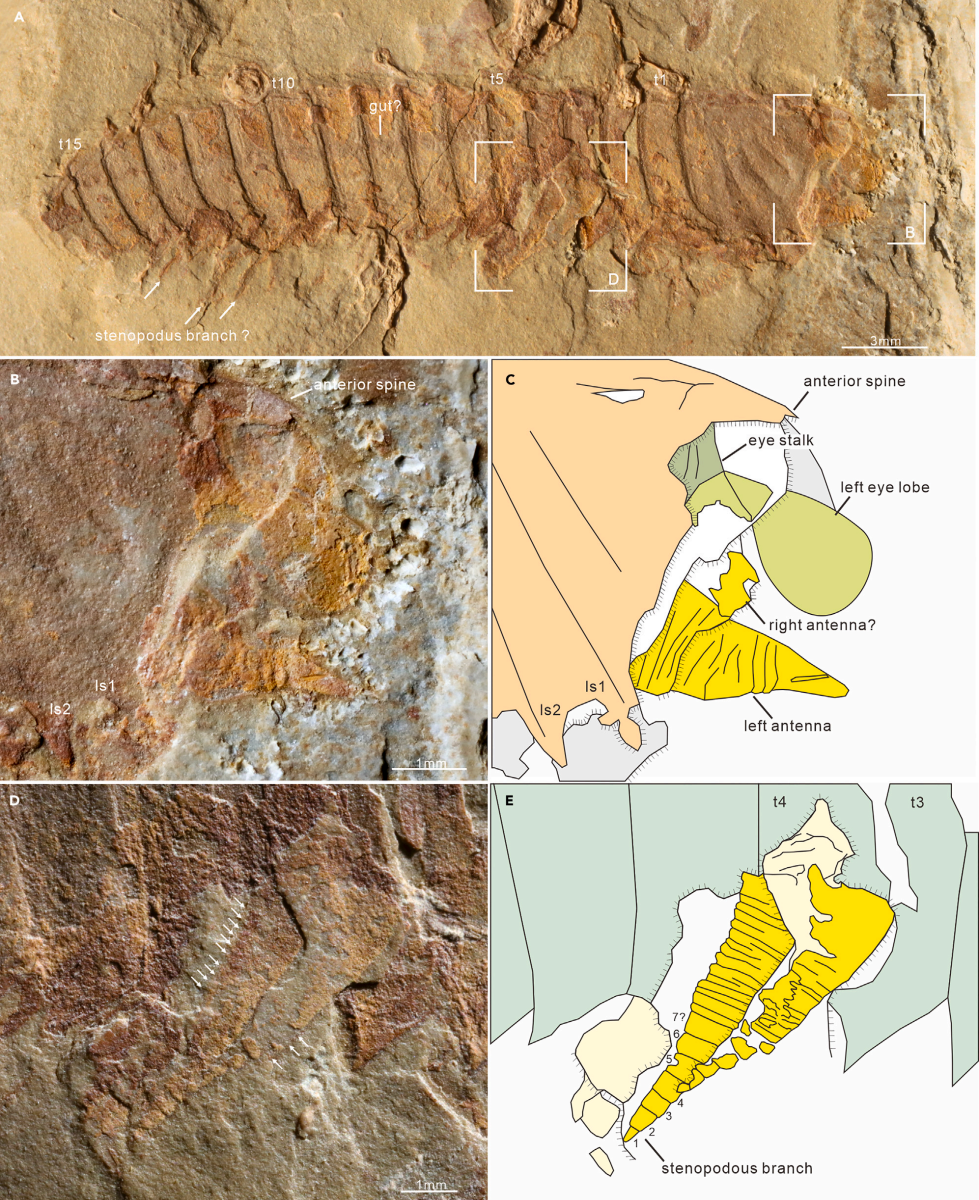
General morpho-anatomy and details of appendicular structures of *Urokodia*, EJ-1563 (A) Lateral view of the entire specimen. (B and C) Enlargement of head region, showing appendicular structures extending beyond the head shield, including a pair of stalked eyes, and an antenna. (D and E) Enlargement of the anterior thorax, showing appendages of the third and fourth segments, characterized by an elongate proximal element articulated with a stenopodous branch consisting of seven podomeres.

**Figure 5:**
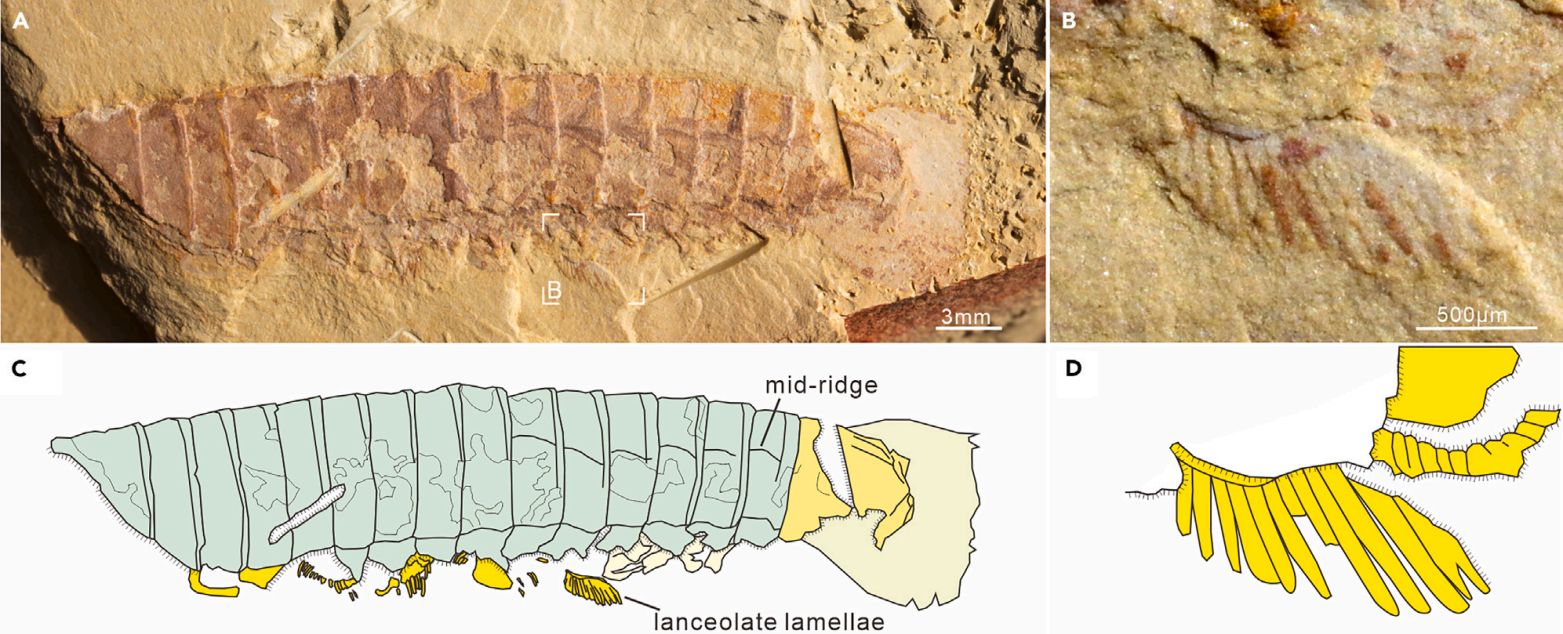
Lanceolate lamellate thoracic appendages of *Urokodia*, SJZ-B23-093 (A) Lateral view of the entire specimen. (B) Close-up of lanceolate lamellae. (C) Camera-lucida drawing of (A). (D) Interpreting morphologies of the trunk and sppendages.

*Head shield*. Nineteen new specimens (6 in dorsal and 13 in oblique views) show features consistent with those described by previous works (Hou et al.^3^^,^^10^^,^^11^ and Zhang et al.^12^): sub-square in dorsal view, 2.80–4.74 mm in sagittal length, vaulted dorsally, without a defined axial region, carrying one pair of anterior spines and three pairs of lateral spines (Figure 1). Lateral spines stretching anteroventrally and corroborated by the fact that they are typically well-defined in laterally compressed specimens (Figures S1A and S1B; Hou et al.,^10^ Pl. Ⅴ, fig. 1; Pl. Ⅵ, fig. 3; 2017, figs. 20.31b and 20.31d; and Zhang et al.,^12^ fig. 4B) but obscure in dorsal view (Figures 1A and S2; Hou et al.,^10^ Pl. Ⅴ, fig. 2; Pl. Ⅵ, figs. 4 and 5). Measurements of the new lateral view specimen SJZ-B08-002 and the oblique specimen HY-020 described in Zhang et al.^12^ indicate that the length of the anterior spines is about 33% of the shield, slightly longer than the lateral spines, accounting for about 26% of shield length (Table S1). Four pairs of ridges are present in the lateral region as described by Hou et al.^10^; the first pair pointing to the notch between the anterior and the first lateral spine, while the succeeding three pairs correspond to the lateral spines (Figures 1, 2A, 2E, S1A, S1B, and S2A). Transverse impressions are observed between adjacent ridges in two new specimens. In the intact specimen JS-1508A, one impression is present between the third and fourth ridge (Figure S2A), whereas in anteriorly deficient specimen JS-1527 two impressions can be observed between the posterior three ridges (Figure S2C), probably hinting segmentation of the head.

*Thoracic exoskeleton*. The number of thoracic tergites was described as 14 by Hou et al.^3^^,^^10^^,^^11^ and 14 or 15 by Zhang et al.^12^ New material including 18 specimens with thoracic tergites countable demonstrates that the thorax has three phenotypes, varying from 13 to 15 in segment number (Figures 2A–2F) (Table S1). Size measurements of new specimens indicate the only known specimen with a 13-segmented thorax is shorter than the specimens with 14 thoracic segments (Figure 2G). Among 16 specimens with 15 thoracic tergites, 12 specimens have a thoracic length longer than the other two phenotypes; the smallest specimen, however, is shorter than the phenotype with 13 thoracic tergites (Figure 2G); additional three specimens show the thorax shorter than the specimens with 14 thoracic tergites (Figure 2G). Re-examination of previously published specimens that were described as having 14 thoracic tergites indicates that the holotype (108311) with unspecified pygidial bears 14 identifiable thoracic tergites (Hou et al.,^10^ Pl. Ⅴ; fig. 1), while specimens 108315 in Hou et al.^10^ (Pl. Ⅵ, fig. 2), YKLP 13933 and YKLP 13934 in Hou et al.^3^ (figs. 20.31b and 20.31d) exhibit 15 thoracic tergites.

All available specimens demonstrate thoracic tergites have a similar morphology in dorsal view, sub-rectangle in outline, overlapping the succeeding one at the posterior margin (Figure 1), equivalent in sagittal length, while tapering gradually toward the rear, reducing approximately 28.36% from the first to the last thoracic tergite (Table S1). Similar to the head shield, thoracic tergites feature a vaulted cross-section, supported by the fact that the pleural spines of tergites are evident in specimens preserved in lateral aspect (Figures 2E and 2F; YKLP 13933 and YKLP 13934 in Hou et al.,^3^ figs. 20.31b and 20.31d), but obscure in dorsoventally flattened specimens (Figures 2A–2D; YKLP 13932 in Hou et al.,^3^ fig. 20.31c). Moreover, the micro-CT scanning data of dorsally compressed thorax of SJZ-B21-078 confirms this feature and reveal pleural spines bend toward the posteroventral side (Figures 3F and 3G). Weak mid-ridges can be observed in the tergites of specimens preserved in dorsal aspect (Figures 1A and 5A), while the ventral ones are only exhibited in the micro-CT scanning of the last two or three posterior thoracic segments (Figures 1C, 3E, and 3I).

**Figure 6:**
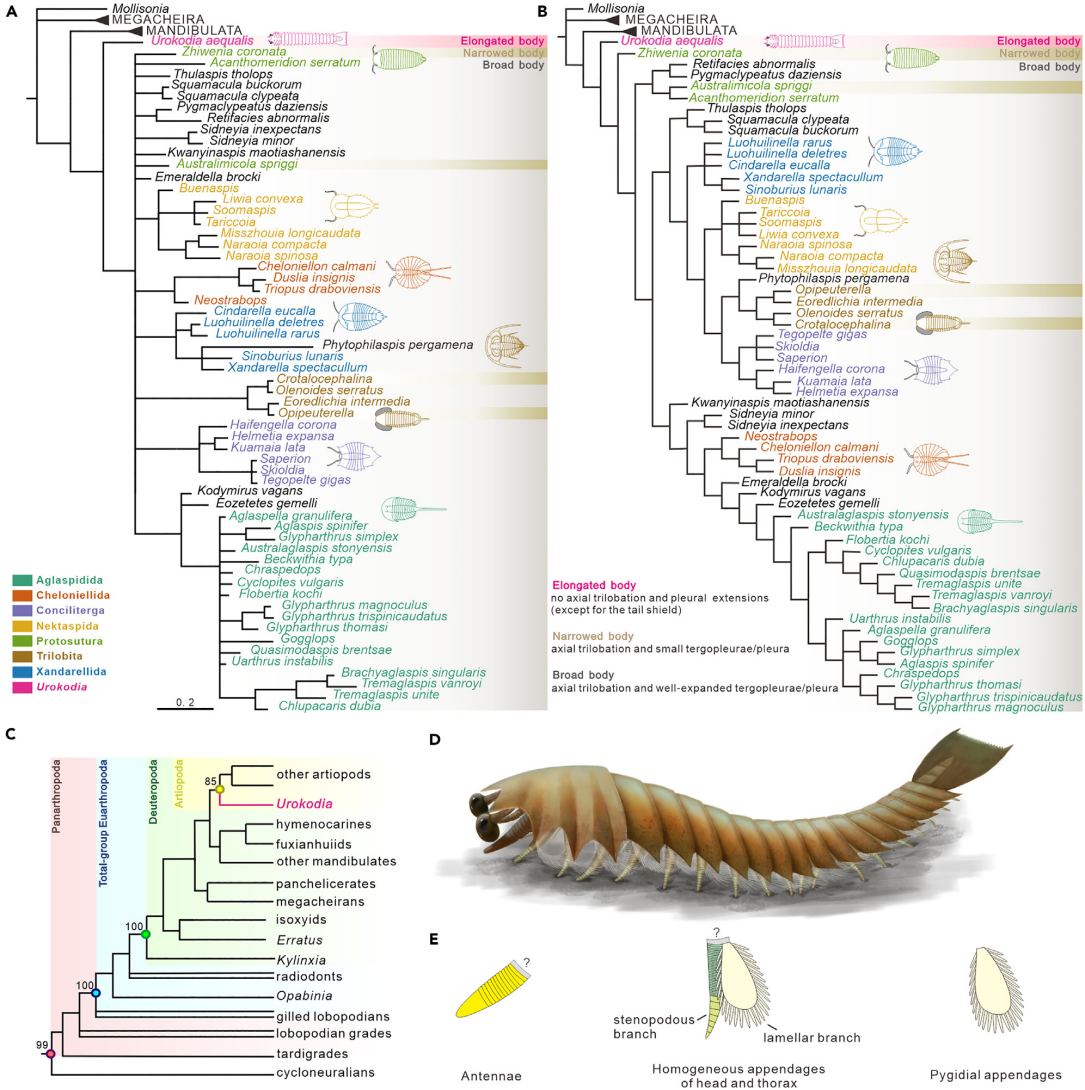
Results of cladistic analyses and the interpretative diagram of *Urokodia* (A and B) Results of the Artiopoda-centric phylogenetic analyses, showing *Urokodia* as a basal branch of the Artiopoda. (A), consensus tree result of Bayesian phylogenetic analysis. (B), equal weights analysis consensus tree from 5 most parsimonious trees. Elongate-, narrow-, and broad-artiopodan body plans are labeled respectively in pink, yellow, and gray. (C) Simplified cladogram of the panarthropod phylogenetic analysis performed by Bayesian phylogenetic inference, revealing *Urokodia* (in pink) as the earliest diverging representative of the Artiopoda. (D) Artistic reconstruction of *Urokodia*. (E) Interpretative drawing of the antennae, the homogenous appendages following antennae, and the pygidial appendages.

*Pygidial exoskeleton*. The length ranges from 3.33 to 5.98 mm and the maximum width at the posterior edge, similar to length (Table S1). New material contains eight specimens showing complete morphology of the pygidial shield, which coincides with the description Zhang et al.^12^ The pygidial shield, dissimilar to the head shield, is dorsoventrally flattened, sub-trapezoid in outline, bearing a pair of larger posterior spines and a series of smaller spines on the straight posterior margin. The axial region is slightly bulged, tapering posteriorly, making up 51.4% length and 43.7% width of the pygidial shield (Figures 1 and S3). Two transverse impressions equally divided the axial region into three parts, mirroring the segmentation of the anxial region (Figures 1 and S3). Re-examination of the holotype (Hou et al.,^10^ Pl. Ⅴ; fig. 1) indicates that pygidial shield is incomplete and unable to support the previous depiction that head and pygidial shields were identical in outline. As for the paratype (108315 in Hou et al.,^10^ Pl. Ⅵ; fig. 2), the line drawing (Hou et al.,^10^; fig. 6) demonstrated the head similar to the pygidial. However, new observation indicates that the pygidial shield of this specimen is incomplete and the supposed pygidial shield is a disarticulated head of another specimen (Figure S4). Additionally, two specimens illustrated in Hou et al.^3^ (figs. 20.31b and 20.31d) show the oblique view of the pygidial shield, which markedly differ from the head shield. In addition, the micro-CT scanning data of SJZ-B21-078 illustrates that the pygidial segments are armed with mid-ridges (Figures 3E and 3I).

*Soft*-*tissues*. Optical observations of 10 specimens, alongside micro-CT scanning data (Videos S1 and S2) from two of them, reveal anatomies of eyes and appendages described as follows.

*Eye*. Eyes can be observed in six specimens, including five new specimens along with the specimen (YKLP 13934) shown in Hou et al.^3^ (fig. 20.31d). A consistent morphology of the eye lobe is evident, although three specimens (two new and YKLP 13934) only display the distal portion. They are characterized by a reniform shape, large size, with a maximum diameter about 1.33 mm and a maximum long axis around 1.98 mm, which accounts for 46.8% length of the head shield (Figures 4A and S5). Eye lobes extend beyond the margin of head shield, protruding from the anterior margin in two specimens (Figures 3A, S5C, and S5D) and from the lateral margin in others (Figures 4A, S1A, S1B, S5A, and S5B; Hou et al.,^3^ fig. 20.31d). Two new specimens show eye lobe attached to a short cylindrical stalk (Figures 4D, 4E, S5A, and S5B). The proximal region of the eyestalk is not visualized in our specimens.

*Antennae*. Zhang et al.^12^ illustrated a linear structure in front of the head shield and tentatively interpreted as a stout antenna. Herein, four additional specimens show the anteriormost appendage, which immediately follows the eye lobe and projects beyond the notch between the anterior and lateral spines (Figures 1C, 3E, 4B, and 4C). In specimen EJ-1563 (Figure 4B), the first appendage is conical in lateral view, 2.13 mm long and 1.91 mm wide at the base, and decorated with closely spaced annulations though exact number of annuli is unable to be determined. Micro-CT data of the two specimens (SJZ-B16-078 and SJZ-B21-078) also demonstrate a similar conical shaped structure in dorsal view (Figures 1C and 3E). The linear structure illustrated in the new specimen MF-1581B (Figure 3C) and the specimen HY-020 reported by Zhang et al.^12^ (Figure S6) is sub-cylindrical and consistent in width. Overall, the anteriormost appendage is a fleshy, flexible, annulated, and non-sclerotized structure without joints.

*Post-antennal appendages*. Specimens revealing post-antennal appendages are sparse. The head of the parallel specimen SJZ-B21-078 exposes the distal portions of three lamellate flaps and the two annulated branches (Figures 3B and 3C). Three flaps are separated by thin layers of muddy layers: the first lobe fragmented distally, the second and third ones showing an arched margin on which fringed with three and six lamellae, respectively. Flapped branches extend posteriorly, each similar in size, with maximum width about 0.70 mm. The proximal region of the two branches is obscured by shield and absent in the distal portion, visible length of the first being 0.78 mm long and 0.41 mm wide, and the second one being 1.06 mm long and 0.49 mm wide. Micro-CT scanning of SJZ-B16-078 reveals three pairs of post-antennal head appendages corresponding to the lateral spines, respectively (Figure 1C), which are homogeneous in shape and composed of a flap-shaped branch fringed with lamellae and a segmented branch with multiple podomeres, three visible in the second and six in the third one.

In laterally compressed specimen EJ-1563, five thoracic appendages have been exposed, which are similar (identical) to post-antennal head appendages in structure, but slightly larger in size. In the third and fourth segments, the proximal portion of the appendages bears a single elongate element with well-developed annuli (at least 12) (Figures 4D and 4E), up to 1.84 mm in length with a maximum basal width of 0.69 mm, and is articulated with a stenopodous branch possibly bearing seven podomeres, tapering distally (Figures 4D and 4E). The distal portion of appendage flaps are well-defined in the eleventh segment of specimen SJZ-B23-093 (Figure 5), with maximum width about 1.20 mm, fringed with closely spaced lamellae, 10–11 per 1 mm, and the width of each lanceolate lamella slightly contracting toward the distal. Tomographic data demonstrate paired appendages of the 1^st^ and 6^th^–11^th^ thoracic segments in specimen SJZ-B16-078, each of which consists of two branches: an outer flap fringed with lamellae and a stenopodous branch (Figure 1C).

Accordingly, post-antennal head and thoracic appendages are considered to be homotypic, consisting of a proximal annulated element articulated with a stenopodous branch with seven podomeres and a flap-shaped lobe fringed with lamellae.

Pygidial appendages have been observed in two specimens by micro-CT scanning, resembling the flapped-shaped branches of thoracic appendages in size and morphology (Figure 3H). In left side of SJZ-B21-078, three lobes are inclined in a consistent way, directing posteriorly and overlapped the succeeding one (Figure 3H). Information on the proximal element or stenopodous branch seen in thoracic appendages is not revealed in these two specimens.

### Phylogenetic analyses

The new data presented in this study, which encompass features of exoskeletons and soft tissues, offer a foundation for examining the phylogeny of *Urokodia* and its allies. In this context, we constructed three cladistic trees (Figure 6) utilizing *Urokodia* from two different datasets: the Atriopoda-centric matrix (Figures 6A and 6B) and the panarthropod phylogenetic matrix (Figures 6C and S8).

Both Bayesian phylogenetic inference and maximum likelihood methods of the Artiopoda-centric analysis assign *Urokodia* within the Artiopoda as a sister taxon to all other artiopods (Figures 6A and 6B). However, the phylogenetic interrelationships of other artiopods in these two analyses are sensitive to the methodology employed. Bayesian analysis resolved other artiopodan taxa as the polytomy, but a clade (*Thulaspis*+*Squamacula*) and Protosutura were failed to recover. Parsimony analysis restored the other artiopods as monophyly, but also failed to restore Protosutura. *Zhiwenia* was supported as the basal branch of this monophyletic group, while the (*Australimicola*+*Acanthomeridion*) and the recently redescribed (*Retifacies*+*Pygmalypeatus*)^13^^,^^14^^,^^15^ were resolved as the sister group. The clade of *Thulaspis* and *Squamacula* was retrieved as the closest groups to the monophyletic group comprising Xandarellida, Nektaspida, Trilobita, and Conciliterga. Newly added *Mollisonia* were consistently reinstated as the polytomy with Megacheria in two analyses. And the new addition of *Opipeuterella* and *Crotalocephalina* were both consistently recovered within Trilobita.

The panarthropod phylogeny was reconstructed using Bayesian phylogenetic inference, displaying largely congruent topologies among the major panarthropod groups (Figure 6C). Among this topology, the position of *Urokodia* is recovered as the basal branch of the Artiopoda. Given that artiopods appear monophyletic with a high posterior probability in this Bayesian inference of the resulting tree (85), we prefer to consider *Urokodia* as early artiopods, distinguishing it from previous analyses that classified it as Mollisoniidae.^10^^,^^12^^,^^16^

## Discussion

### Phenotypes

*Urokodia* was previously described as having 14 or 15 thoracic tergites.^10^^,^^12^ New observation indicates the thorax consists of 13–15 tergites (Figure 2). Measurements demonstrate the sagittal length of the thorax generally increasing with the number of thoracic tergites from 13 to 15 (Figure 2G). Such intraspecific variation seen in fossil arthropods is commonly interpreted as a series of growth stage, as observed in trilobites,^17^^,^^18^ fuxianhuiids,^19^ and “Orsten” type crustaceans.^20^ Remarkably, the smallest specimen (JS-1546) carries a 15-segment thorax. This measurement result is reminiscent of euarthropods that possess intraspecific variation in the number of segments, such as *Chuandianella*. Measurements of this species show that the smallest specimen is the one with the maximum number of abdominal segments (the seven-segmented phenotype).^21^ Hence, the variation in the number of thoracic segments of *Urokodia* also can be interpreted an intraspecific polymorphism.

### Affinities

*Urokodia* was previously assigned to the Mollisoniidae,^10^^,^^12^^,^^16^ based on sharing similar dorsal exoskeleton. However, new morphological and phylogenetic results do not support that assignment (Figures 6A and 6B). Key differences are manifested in appendages and pygidial shield. The anteromost appendages of *Urokodia* revealed in two specimens (Figures 4B and S6) and in micro-CT data of SJZ-B16-078 (Figure 1C; Video S1) are short, flesh antenniform rather than chelicerae-form as seen in *Mollisonia*. Micro-CT scanning demonstrates that the megacheiran-like artifact was derived from overlying pyritized outline of the appendages (Figure 3E; Video S2). The post-antennal appendages of *Urokodia* consist of an outer flap fringed with lamellae and a stenopodous branch, whereas *Mollisonia* bears uniramous cephalic limbs and proto-book gills on the thorax.^22^ The homomorphic lamellate flaps of the *Urokodia*, especially pygidial appendages consisting only of lamellar lobes, are superficially reminiscent of the main branch of the proto-book gills in *Mollisonia*. However, no additional lobes were revealed in our specimens, and the marginal lamellae of *Urokodia* are characterized by a long lanceolate shape (Figure 5C), as is characteristic of exopods in artiopods, such as those of *Emeraldella*^23^ and *Luohuilinella*.^24^

In addition, new data reveal morphological differences of the two taxa in dorsal exoskeleton. The head shield of *Urokodia* differs from that of *Mollisonia* in its complex marginal structures including one pair of anterior spines and three pairs of lateral spines. Unlike *Mollisonia* that bears well-developed sub-triangular pleurae in thoracic tergites,^22^ *Urokodia* lacks pleurae in thorax. Critically, the pygidial shield of *Urokodia* bears a well-defined, segmented axial region that sets it apart from that of *Mollisonia*. Our phylogenetic results support *Urokodia* with an affiliation of the Artiopoda, as the basal most branch outside the main subclades (Figures 6A and 6B). Thus, we assign *Urokodia* to the Artiopoda rather than the Mollisoniidae.

### Origin of the artiopodan body plan

In artiopods, typically trilobitomorphs, tagmatization is characterized by a tripartite body division, comprising a head, a thorax, and a pygidial longitudinally, along with an axial lobe and two lateral lobes transversely. Compared with typical artiopods, e.g., trilobites,^25^ *Urokodia* exhibits an elongate body without lateral lobes in head or thorax. This recalls the narrow body plan of artiopodan representatives such as *Zhiwenia*^26^ and *Australimicola*^27^ with a poorly defined axial region. Nevertheless, the extended tergopleurae can be observed in the trunk of these taxa. Additionally, artiopods are mostly dorsoventrally flattened, while the body of *Urokodia* strongly vaults in the anterior half and becomes flat toward the rear in most specimens. Intriguingly, the pygidum of *Urokodia* is dorsoventrally flat and exhibits a segmented axial region resembling the pygidium of many artiopods (Figures 1A, 3A, and 5A).

New data demonstrate that the head organization of *Urokodia* corresponds to that of major artiopodan clades,^28^^,^^29^^,^^30^ typified by the presence of three pairs of post-antennal appendages. Although two pairs of post-antennal head appendages of *Thulaspis* were remarked to represent a possible ancestral state of artiopods,^13^ the three or four appendicular configuration is widespread among the deep branches, such as three pairs in *Zhiwenia* and *Acanthomeridion*,^26^^,^^31^ and four pairs in *Retifacies*^14^ and *Pygmaclypeatus*.^15^ Moreover, the credible evidence for the fact that only two pairs of post-antennal appendages develop on the head of *Thulaspis* is still needed, since this finding can be confirmed only by the extension of exopods beyond the head shield and the protopodites imprints on the head.^13^

Comparably, stalked anteroventral eyes are widely associated with euarthropods, and are markedly distinct from the sessile dorsal eyes commonly found in some members of Artiopoda, particularly trilobites^32^ and aglaspidids.^33^ However, recent investigations have shown evidence for the presence of stalked ventral eyes accommodating in the anterolateral notch of the head shield among numerous taxa of non-trilobite artiopods, such as *Kwanyinaspis*,^34^ *Luohuilinella*,^35^^,^^36^ and *Zhiwenia*,^26^ allowing for a favorable comparison with *Urokodia*. More critically, the dorsal penetrating eyes of *Sinoburius*, *Xandarella,* and probably *Phytophilaspis* have been confirmed as ventral stalked eyes,^35^ suggesting that ventral stalked eyes may be more widespread in artiopods.

The first appendage of *Urokodia* significantly differs from the long, multisegmented ones of other artiopods by its fleshy nature and short length. Such a feature evokes the primary antennae of onychophorans, like the extant taxon *Euperipatoides rowelli* (Ou et al.,^37^; figs. 3b–3e), equipped with a pair of rod-like fleshy antennae. However, the first appendage of *Urokodia* has inserted ventrally into the second segment of the head, posterior and adjacent to the stalked eyes (Figures 4A–4C). In this context, it seems likely that this fleshy appendage is not homologous to the primary antennae of onychophorans, given that its position, including the arose segment and dorsoventral orientation. Similar antennae have not been reported in other euarthropods and their homology remains open.

Post-antennal appendages of *Urokodia* offer a high comparability with those reported in artiopods. Firstly, *Urokodia* shares similar flap-like lamellae lobes with many non-trilobite taxa such as *Zhiwenia*,^26^ *Thulaspis*,^13^ and *Squamacula*.^38^ Secondly, limb heteronomy occurs in *Urokodia*. The appendicular divergence along the antero-posterior body axis is manifested in *Urokodia* by its pygidial appendage heteronomy. Similar appendage differentiation was also reported in the artiopod *Emeraldella brutoni*. Apart from the differentiation of the first post-antennal appendages into a segmented uniramous branch, its terminal segment only carries lamellar flap.^23^ Thirdly, neither endites nor differentiation between proximal to distal podomeres have been observed in in the stenopodous branch of *Urokodia*. Such simple limb reconstruction was also reported in *Zhiwenia*^26^ and *Luohuilinella*^24^^,^^35^ and considered as an ancestral state for the Artiopoda.

A broad body plan with well-expanded tergopleurae or pleurae is widespread among artiopods (Figures 6A and 6B). However, a narrow version of the body configuration is also present in *Zhiwenia*^26^ and *Australimicola*,^27^ as well as trilobites *Opipeuterella*^39^ and *Crotalocephalina*.^40^ Notably, the narrow body of these taxa is attributed to having a small tergopleurae/pleurae, distinguishing them from *Acanthomeridion* that has narrow body because of ventral curvature of the pleura.^31^ *Urokodia* is unique in its head shield and thoracic tergites lacking pleural lobes while its pygidial shield with a well define axial region. Placing this uniqueness into the phylogenetic context would broaden insight into body plan diversification of this clade. Although no unified understanding of the internal relationships of the Artiopoda has arose from our results or previous investigations,^13^^,^^14^^,^^15^^,^^26^^,^^28^^,^^31^ *Urokodia* are consistently reverted as the deepest branch in our cladistic analyses (Figures 6A and 6B), which suggests an ancestral body plan for this clade. As an approximate ancestral artiopodan, *Urokodia* informs a possible origin schema for the artiopodan body. The trilobate body may have originated initially from the pygidial shield.

### Convergence of elongate body plan

Drawing on the fossil records as well as morphological investigations of extant forms, elongate body plan has been documented across arthropods lineages. For instance, the lobopodian body plan is characterized by cylindrical, elongate type, e.g., in the early Cambrian *Collinsium ciliosum*^41^ and *Onychodictyon ferox*^37^ from southern China. For the stem and crown groups of the Euarthropoda, numerous representatives also exhibit an elongate body, but tapering posteriorly, such as radiodonts,^42^^,^^43^ *Kylinxia*,^44^ megacheirans,^45^^,^^46^ fuxianhuiids,^47^^,^^48^ the major clades of mandibulates (e.g., Cambrian hymenocarines; extant remipedes and anostracans),^49^^,^^50^^,^^51^ and some Chelicerata representatives (e.g., *Mollisonia* and *Habelia*).^22^^,^^52^ *Urokodia* provides an elongate case in Artiopoda. It seems that the elongate body pattern is widespread across arthropod lineages, which may arise convergently multiple times among arthropods lineages.

### Conclusion

A comprehensive morphological investigation of *Urokodia* revealed this taxon is featured by a suit of new traits, including a head shield with transverse impressions, a trimorphic thorax with 13–15 tergites, a trilobated pygidial shield, one pair of ventral stalked eyes, a pair of antenniform first appendages, and a series of post antennal appendages with lamellar lobes. Cladistic analysis revised *Urokodia* witjin the basal position of the Artiopoda, providing a unique body plan of this clade, an elongate vaulted head-thorax region without pleural extensions followed by a flat pygidial shield with a well define axial region. As an approximate ancestral artiopodan, *Urokodia* provides new information for understanding the origin of the Artiopoda body plan and raises a hypothesis that trilobatization possibly incepted from the pygidial shield. With the new data, combined with the widespread presence of the elongated body plan across major lineages of arthropods, we tentatively proposed that elongate body plan evolved convergently among arthropods lineages.

### Limitations of the study

Fossils used in our study from five localities of the Chengjiang biota, i.e., Erjie, Haoyicun, Jianshan, Mafang, and Sanjiezi (EJ, HY, JS, MF, and SJZ, respectively) are unlikely represent a single population in strict sense, which is always the case in paleontological studies. However, in our study, all specimens were collected from a narrow stratigraphical range of the *Eoredlichia*-*Wuyangaspis* trilobite biozone and are generally considered to reflect the size and morphological range of “a single population.”

## STAR★Methods

### Key resources table

**Table:** 

REAGENT or RESOURCE	SOURCE	IDENTIFIER
**Deposited data**		

Raw scanning data of Micro-CT of SJZ-B16-078	Mendeley Data Liu et al. Fig. 1 (Video S1)	https://data.mendeley.com/preview/t7vh977fz8?a=003c66db-260e-48c8-b49f-1eb2e19e7bb1
Measurement data of *Urokodia* body length	Mendeley Data Liu et al. Fig. 2 (Table S1)	https://data.mendeley.com/preview/t7vh977fz8?a=003c66db-260e-48c8-b49f-1eb2e19e7bb1
Raw scanning data of Micro-CT of SJZ-B21-078	Mendeley Data Liu et al. Fig. 3 (Video S2)	https://data.mendeley.com/preview/t7vh977fz8?a=003c66db-260e-48c8-b49f-1eb2e19e7bb1
Atriopoda-centric phylogenetic datasets	Mendeley Data Liu et al. Fig. 6 (Table S2)	https://data.mendeley.com/preview/t7vh977fz8?a=003c66db-260e-48c8-b49f-1eb2e19e7bb1
Panarthropod phylogenetic datasets	Mendeley Data Liu et al. Fig. 6 (Table S3)	https://data.mendeley.com/preview/t7vh977fz8?a=003c66db-260e-48c8-b49f-1eb2e19e7bb1

**Software and algorithms**		

CorelDraw X9	This paper	https://www.coreldraw.com/
Adobe Photoshop CC	This paper	https://www.adobe.com/
ImageJ 1.8.0	This paper	https://imagej.nih.gov/ij/
ORS Dragonfly v4.1.7	This paper	https://www.theobjects.com/dragonfly/index.html
MrBayes v3.2.6	This paper	https://nbisweden.github.io/MrBayes/download.html
TNT1.5	This paper	https://www.lillo.org.ar/phylogeny/tnt/

### Resource availability

#### Lead contact

Further information and requests for data should be directed to and will be fulfilled by the lead contact, Xingliang Zhang (xzhang69@nwu.edu.cn).

#### Materials availability

This study did not generate new unique materials.

#### Data and code availability

• Data: All relevant data, including two raw Micro-CT scanning data (Videos S1 and S2), the measurement data of *Urokodia* body length (Table S1), as well as two phylogenetic matrixes (Table S2 and S3) have been deposited at Mendeley Data and is publicly available as of the date of publication. Links are listed in the key resources table.
• Code: This paper does not report original code.
• Any additional information required to reanalyze the data reported in this paper is available from the lead contact upon request.

### Experimental model and study participant details

In this study, the research object is fossil, and no experimental models.

### Method details

#### Materials

A total of 30 specimens of *Urokodia* were observed in this study (29 new specimens, and one previously mentioned by Zhang et al.^12^), which are deposited in the Shaanxi Key Laboratory of Early Life and Environments (LELE), Northwest University, Xi’an, China.

#### Methods

##### Optical imaging

All specimens were observed using stereomicroscopes and photographed by a Canon EOS 5D Mark Ⅱ digital camera equipped with Canon MP-E 65mm 1-5x macro-lens under an incandescent lamp. Camera lucida drawings were made using a Nikon SMZ 100 stereomicroscope and prepared with CorelDraw X9. All images were processed in Adobe Photoshop CC.

##### Micro-computed tomography (Micro-CT)

Eleven specimens with structures potentially hidden beneath the exoskeleton and in the matrix were selected for the micro-CT scanning. All specimens were quickly scanned using Phoenix V Tome X M, and two of them were selected for high-fidelity scanning by Zeiss X-Radia 520 Versa. Scanning pixel sizes of these two finely scanned data was 5.78μm and 8.54μm, respectively. Data were processed using Dragonfly v4.1.7 to generate a 3-D reconstruction of each fossil.

#### Phylogenetic analyses

The Atriopoda-centric phylogeny final matrix contained 71 taxa and 95 characters (Table S2), updated by the previous study in Berks et al.^13^ The character matrix used for the panarthropod phylogenetic analysis updated by a previous study on Zeng et al.,^44^ consists of 84 taxa and 286 characters (Table S3). The phylogenetic matrix used for the phylogenetic analysis consists of. Phylogenetic analyses were performed using Bayesian analyses, and maximum parsimony. Bayesian inference was performed by MrBayes v3.2.6, using default priors and Markov chain Monte Carlo settings for two independent runs of 50,000,000 Markov chain Monte Carlo generations and four chains each under the Mkv + Γ model for discrete morphological character data. Trees were sampled every 1,000 generations in each run, and the first 25% of samples were discarded as burn-in. A maximum parsimony analysis was conducted using the New Technology Search in TNT1.5. This analysis is made for Driven Search with a minimum tree length of 100 searches and the Collapse of lineage tree after each search, under equal character weighting. Before running, Sectorial Search, Ratchet, Drift, and Tree fusing are activated (producing a strict consensus).

### Quantification and statistical analysis

Nineteen specimens were used for morphological measurements which were performed on images using ImageJ v1.8.0. In this study, 11 measurements were documented, including the length of head shield (num. 14), anterior and lateral spines of head shield (num. 2), thoracic tergites (num. 19), pygidial shield (num. 9), eye lobe (num. 3), and antenna (num. 2); the distance between adjacent annuli on the antenna (num. 1); the diameter of eye lobe (num. 3); and the width of head shield (num. 14) and pygidial shield (num. 9).

## Supplemental information

Document S1. Figures S1–S8 and Data S1

Table S1. Measurement data of *Urokodia* body length, related to Figure 2

Table S2. Atriopoda-centric phylogenetic matrixes, related to Figure 6

Table S3. Panarthropod phylogenetic matrixes, related to Figure 6
