# Supplementary material for: Cambrian euarthropod Urokodia aequalis sheds light on the origin of Artiopoda body plan
Source: iScience. 2024 Jul 11;27(8):110443. doi: 10.1016/j.isci.2024.110443 (PMC11325232; doi:10.1016/j.isci.2024.110443)
Supplement: Document S1. Figures S1–S8 and Data S1 [file mmc1.pdf]

iScience, Volume *27*

Supplemental information

Cambrian euarthropod *Urokodia aequalis* sheds light on the origin of Artiopoda body plan

Cong Liu, Dongjing Fu, Yu Wu, and Xingliang Zhang

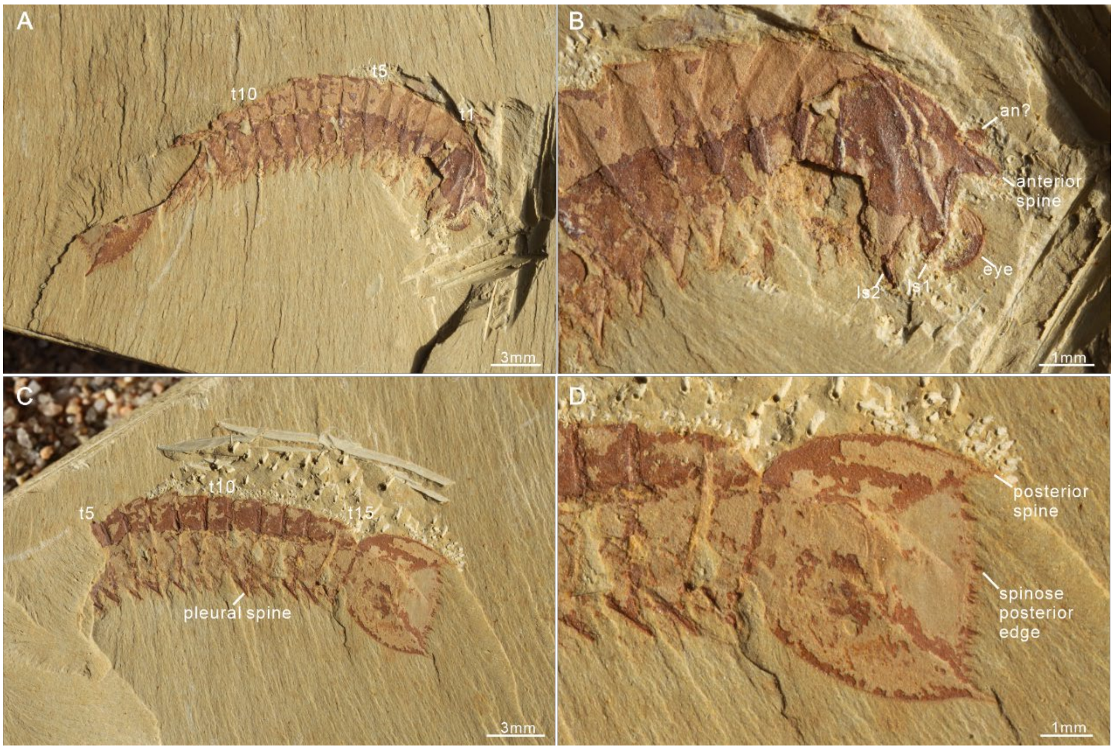

Figure S1. The dissimilarity of head and pygidial shield, related to the STAR Methods. A and B, EJ-1561A, head shield in lateral view showing anterior spine and two lateral spines; eye lobe protruding from the notch between the anterior and the first lateral spines. C and D, EJ-1561B, pygidial shield in dorsal view showing a pair of posterior spines and spinose posterior edge. an, antenna; t, thoracic segment.

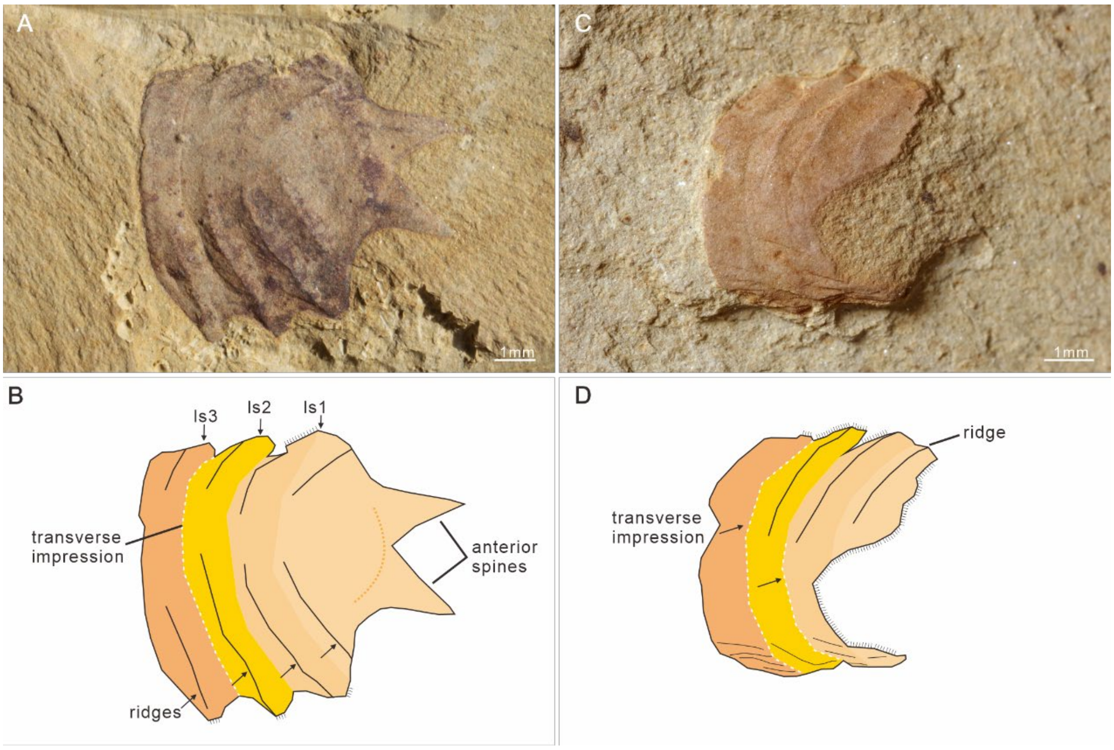

Figure S2. Details of head shield, related to the STAR Methods. A, JS-1508A, head shield in dorsal view, showing a pair of anterior spines, three paired of lateral spines, four pairs of ridges on lateral field, and a transverse impression between the third and fourth ridges. C, JS-1527A, incomplete head shield, showing two transverse impressions in the posterior head shield. B and D, camera-lucida drawings of *A* and *C*, respectively. ls, lateral spines of head shield.

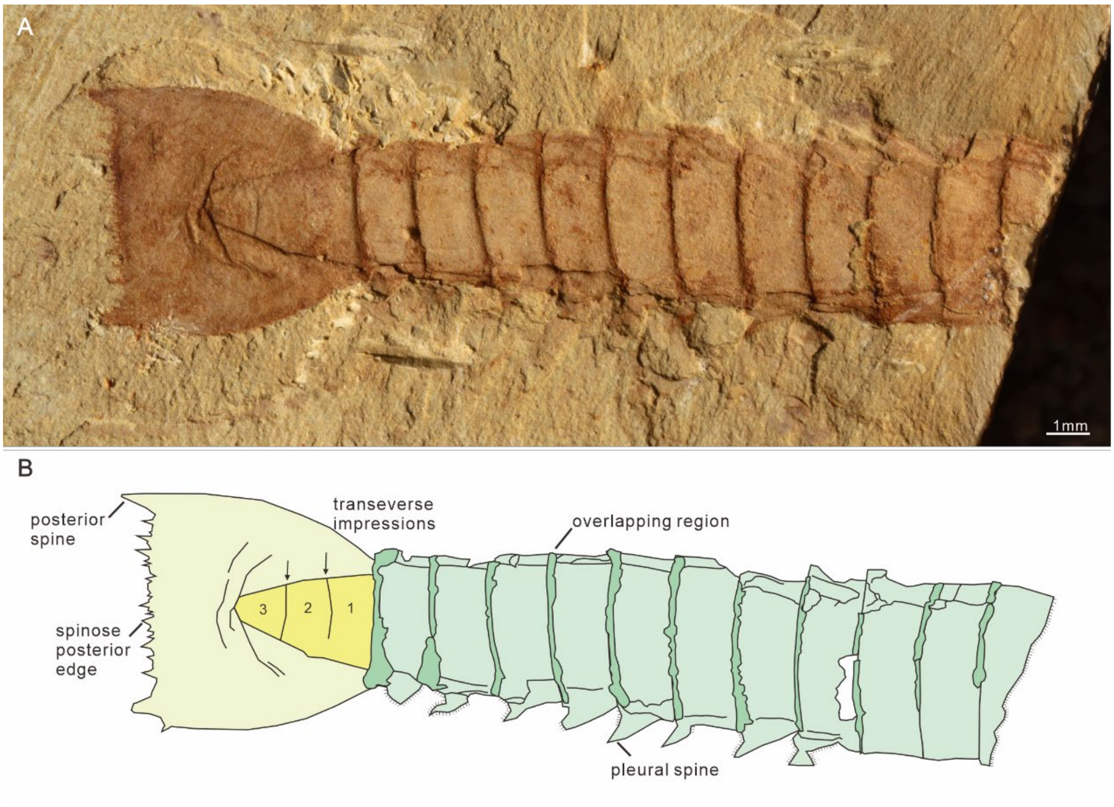

Figure S3. Pygidial shield with segmented axial region, related to the STAR Methods. A, specimen EJ-1564 in dorsal view, showing the pygidial shield trapezoid in outline, with a pair of posterior spines, a straight spinose posterior margin, and the axial region bulged dorsally and separated by two transverse impressions into three segments. B, camera-lucida drawing of *A*.

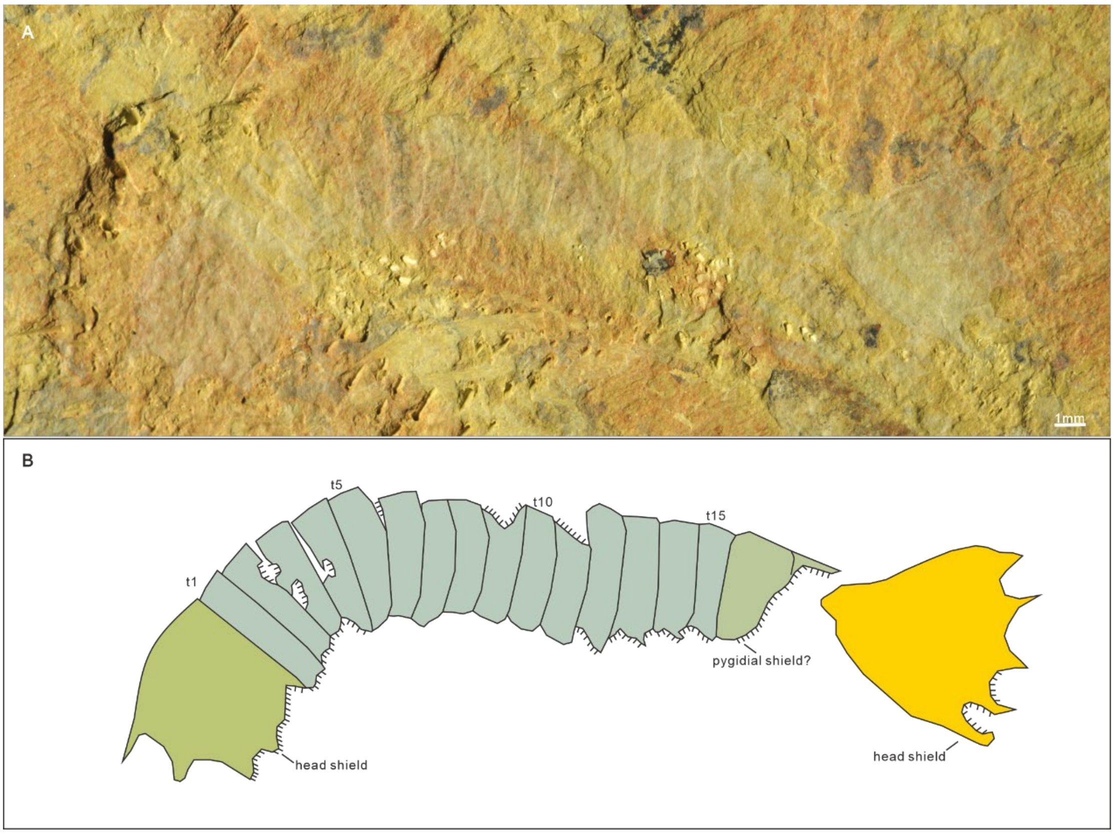

Figure S4. Re-illustration of the paratype 108315, related to the STAR Methods. A, specimen illustrated in Hou et al.[S1], showing two individuals, one in lateral view with a head shield featuring a pair of anterior spines, poorly preserved lateral spines, 15 thoracic tergites, and incomplete pygidial shield; the other showing a head shield only. B, camera-lucida drawing of *A*.

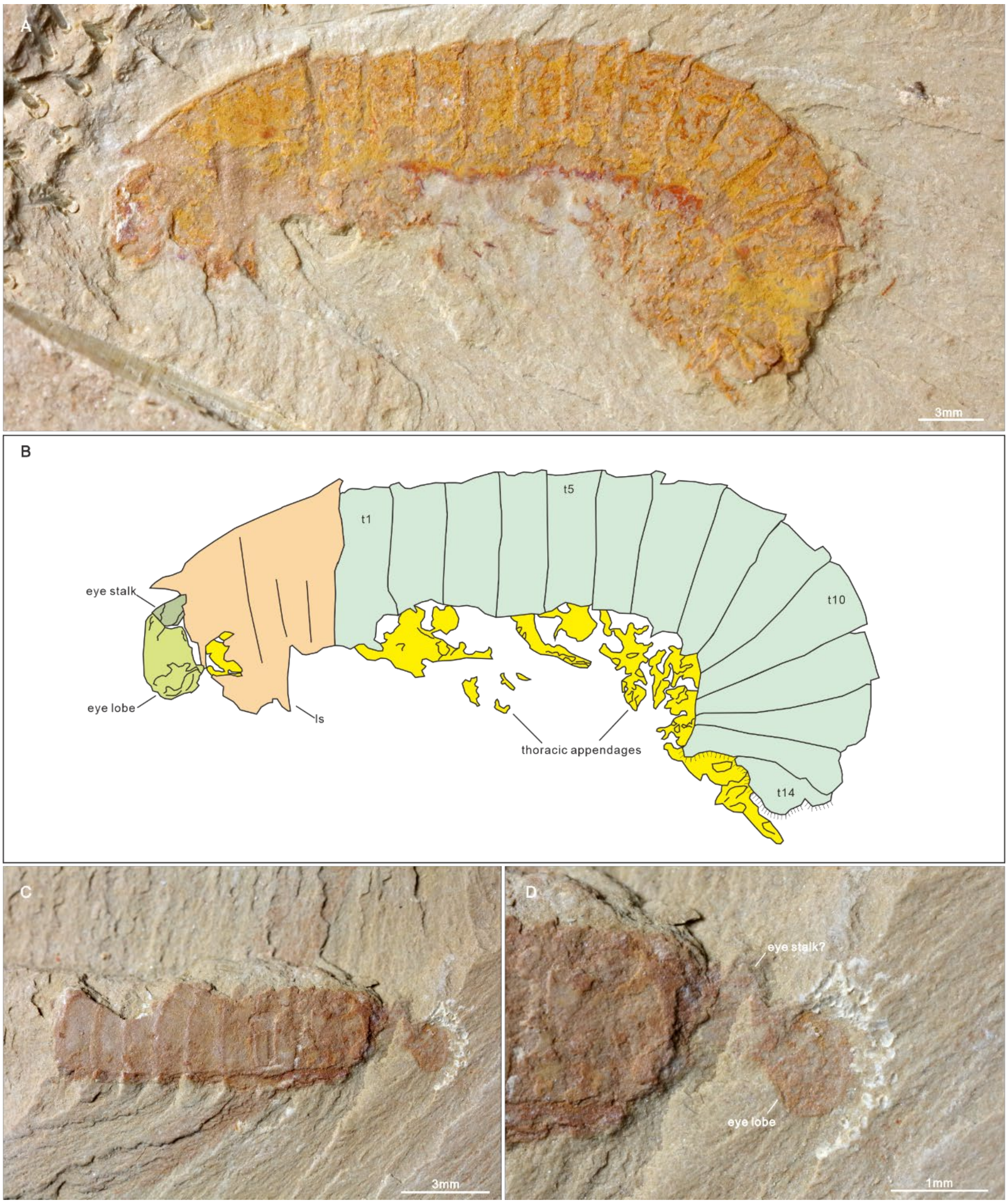

Figure S5. Orientation of eye lobe, related to the STAR Methods. A, SJZ-B01-001A, lateral view, showing stalked eyes protruding beyond the head shield and bending ventrally. B, camera-lucida drawing of *A*. C, general view of the incomplete specimen JS-1557B. D, close-up of the head region of *C*, showing a stalked eye protruding from the anterior margin of head shield.

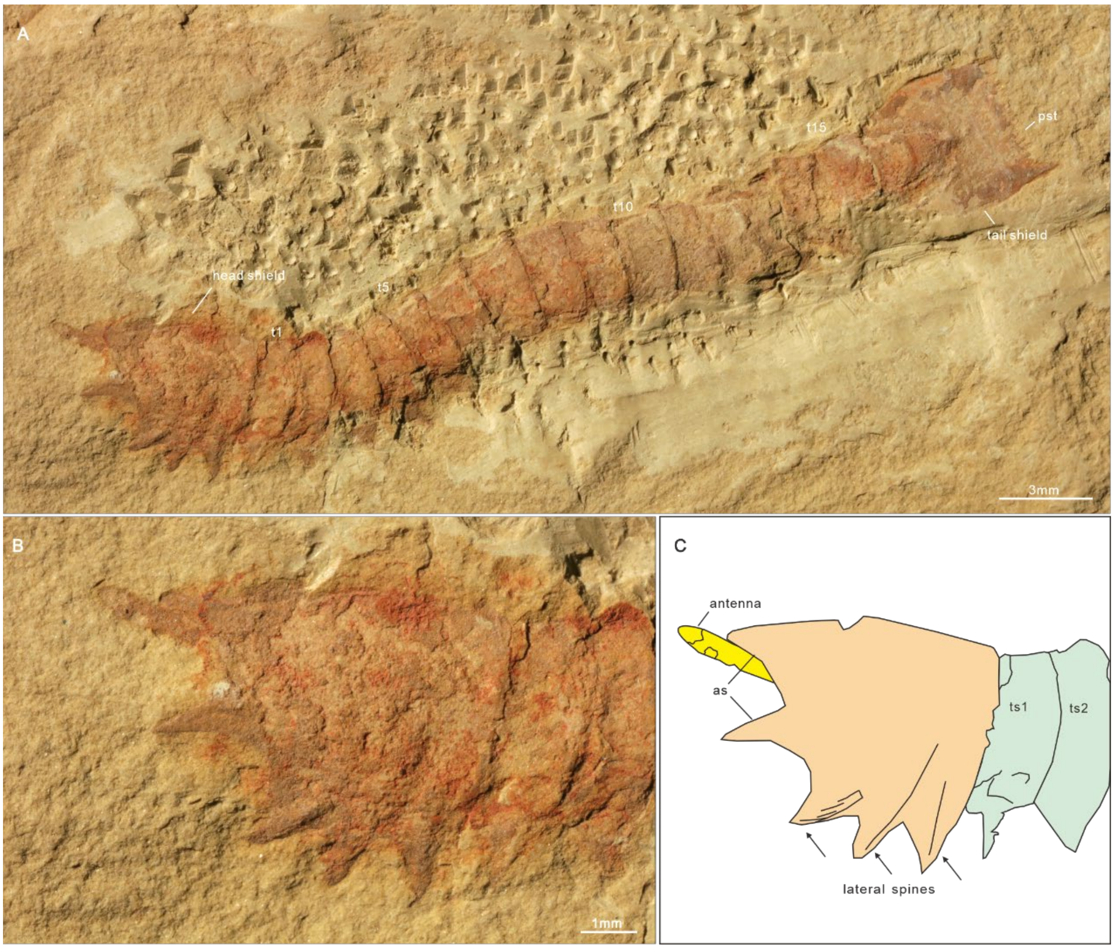

Figure S6. Re-illustration of specimen HY-020, related to the STAR Methods. A, specimen illustrated in Zhang et al. [S2], showing fleshy right antenna, 15 thoracic tergites with axial region. B, enlargement of head in *A*, showing a subcylindrical antenna without joints. C, camera-lucida drawing of *B*.

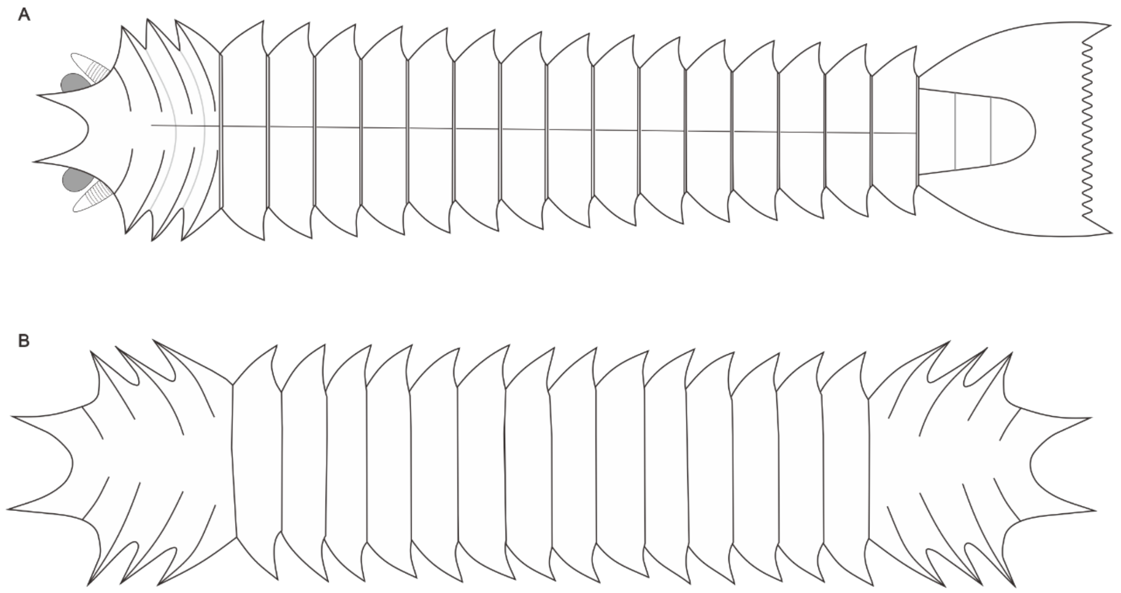

Figure S7. Comparison of new interpretation with previous reconstruction in dorsal view, related to the STAR Methods. A, revised interpretation based on new observations, showing two transverse impressions on the head shield, 15 thoracic tergites, and the dissimilarity of head and pygidial shield. B, re-drawing of the reconstruction in Hou et al. [S1] (fig 10).

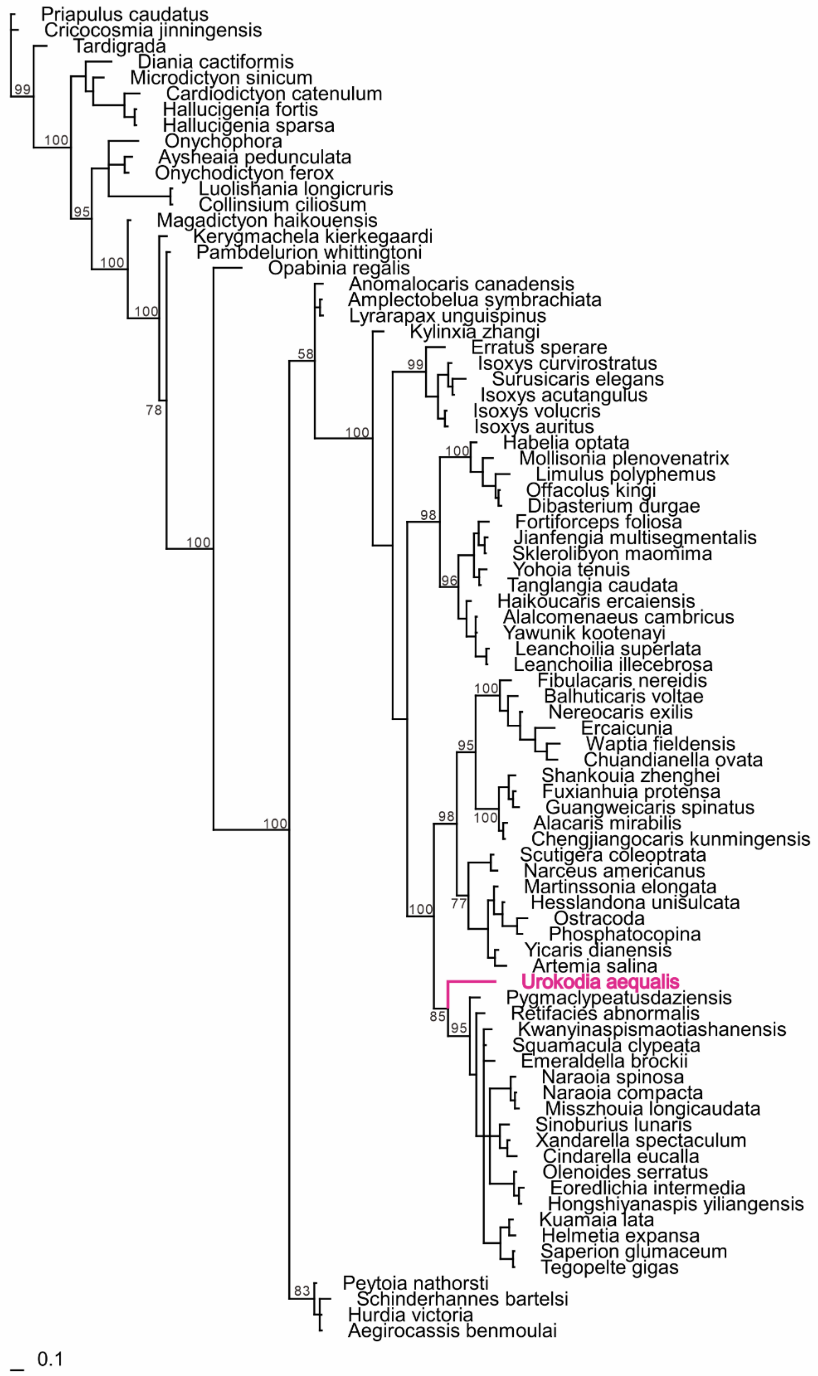

Figure S8. Result of the panarthropod phylogenetic analysis performed by Bayesian phylogenetic inference, related to Fig. 6. Showing *Urokodia* within the Deuteropoda as the basal branch of the Artiopoda with a high posterior probability (85). Nodal supports for the posterior probabilities. Scale bar to indicate expected changes per site.

Data S1: Character list of two phylogenetic analyses, related to Fig. 6.

Atriopoda-centric phylogeny

The character matrix from Berks et al.[S3] was used in the phylogenetic analyses, with *Mollisonia*[S4], *Opipeuterella*[S5] and *Crotalocephalina*[S6] added to form a matrix composed of 71 taxa and 95 characters.

New added morphological characters.

94. Sclerotization of the first appendage: (0) absent; (1) present

    Remark. A feature for *Urokodia*.

95. Relative number of podomeres or annuli in the first appendages compared to that in trunk endopods or legs: (0) close; (1) significantly more; (2) significantly less.
96. Proximal-part of trunk endopodite with multiple podomeres or annuli: (0) absent; (1) present.
97. Maximum number of trunk endopodite podomeres: (0) ≥ 13; (1) 9–12; (2) 8; (3) ≤ 7.

Panarthropod phylogeny

The following list is taken from the Zeng et al. [S7] and includes the modifications made for this study as well as those from Schmidt et al. [S8]. Character headings refer to the original publication by [ZX] and [SX], where X is the corresponding number for that character in Zeng et al. (2020) and Schmidt et al. (2021), respectively.

Cuticle

1. Annulation of integument on main body [Z1]: (0) absent; (1) present.
2. External metameric boundaries on main body [Z2]: (0) invisible or ambiguous; (1) visible and delimited.
3. Sclerotization of main body [Z3]: (0) absent; (1) present.
4. Arthrodization of main body [Z4]: (0) absent; (1) present.
5. Integumental sclerites or nodes on head [Z5]: (0) absent; (1) present.

6. Integumental sclerites or nodes on trunk [Z6]: (0) absent; (1) present.
7. Integumental sclerites with extended reticulated plate-like base [Z7]: (0) absent; (1) present.
8. Integumental sclerites with prominent spine [Z8]: (0) absent; (1) present.
9. Integumental sclerites, relative height to trunk diameter [Z9]: (0) shorter or comparable; (1) at least 1.5times longer.
10. Integumental sclerites, more than two elements per metamere [Z10]: (0) absent; (1) present.
11. Integumental sclerites, paired spines per metamere [Z11]: (0) absent; (1) present.
12. Integumental sclerites, size change along body axis [Z12]: (0) close; (1) anteriormost and posteriormost ones smaller, intermediate ones larger.
13. Papillae or other cuticular derivatives on trunk annuli [Z13]: (0) absent; (1) present.
14. Tergites [Z14]: (0) absent; (1) present.
15. Sternites [Z15]: (0) absent; (1) present.
16. Pleurites [Z16]: (0) reduced or fused; (1) developed.
17. Cuticle biomineralized [Z17]: (0) absent; (1) present.

Eyes

18. Eyes [Z18]: (0) absent; (1) present.
19. Ocelli as primary eyes [Z19]: (0) absent; (1) present.
20. Compound eyes [Z20]: (0) absent; (1) present.
21. Eye stalks [Z21]: (0) absent or very reduced; (1) present.
22. Anteroposterior position of compound eyes in head [Z22]: (0) anteriormost; (1) anterior; (2) middle; (3) posterior.
23. Compound eyes accommodated by dorsal bulge on fused head shield [Z23]: (0) absent, (1) present.
24. Eye stalks of compound eyes covered by fused head shield [Z24]: (0) absent; (1) present.
25. Eye stalks of compound eyes incorporated into fused head shield, forming eye ridges [Z5]: (0) absent; (1) present.

26. Compound eyes with eye slits [Z26]: (0) absent; (1) present.
27. Compound eyes bounded by suture on fused head shield [Z27]: (0) absent; (1) present.
28. Lenses calcified [Z28]: (0) absent; (1) present.
29. Median eyes [Z29]: (0) absent; (1) present.
30. Number of median eyes [Z30]: (0) one; (1) two; (2) three; (3) at least four.
31. Four or five eyes arranged in a sub-transverse band across head shield [Z31]: (0) absent; (1) present.
32. Five compound eyes with size differentiation [Z32]: (0) absent; (1) present.
33. Relative diameter of compound eyes compared with length of bivalved carapace [Z33]: (0) $< 5\%$; (1) 5–10%; (2) $> 10\%$.

Oral structures

34. Position of mouth opening [Z34]: (0) terminal; (1) ventral.
35. Orientation of mouth opening [Z35]: (0) anterior; (1) ventral; (2) posterior.
36. Telescoping feeding apparatus [Z36]: (0) absent; (1) present.
37. Circumoral structures in a radial arrangement [Z37]: (0) absent; (1) present.
38. Differentiation of elements in the outer ring of circumoral structures [Z38]: (0) absent; (1) present.
39. Number of inner spines on the elements in the outer ring of circumoral structures [Z39]: (0) absent or single; (1) multiple.
40. Sclerotized circumoral plates in a radial arrangement [Z40]: (0) absent; (1) present.
41. Sclerotized circumoral plates forming oral cone [Z41]: (0) absent; (1) present.
42. Symmetrical pattern of sclerotized circumoral plates [Z42]: (0) 'triradial'; (1) 'tetraradial'.
43. External surface of sclerotized circumoral plates [Z43]: (0) smooth or bearing weak nodes; (1) bearing prominent scale-like nodes.
44. Furrowed folds on sclerotized circumoral plates [Z44]: (0) absent; (1) present.
45. Labrum [Z45]: (0) absent; (1) present.
46. Hypostome [Z46]: (0) absent; (1) present.

47. Hypostome attachment [Z47]: (0) wide attachment, with or without suture; (1) natant; (2) narrow overlap with pre-hypostome; (3) narrow attachment at hypostomal suture.
48. Hypostome accommodating antennae and extensively covering the mouth [Z48]: (0) absent; (1) present.
49. Hypostome butterfly-shaped [Z49]: (0) absent; (1) present.

Digestive system

50. Gut morphology [Z5]: (0) simple and straight; (1) bearing a series of digestive diverticula or glands.
51. Gut, triangular lateral extension into trunk appendages [Z51]: (0) absent; (1) present.
52. Branching digestive diverticula in head [Z52]: (0) absent; (1) present.
53. Anus opening, position [Z53]: (0) terminal; (1) ventral.

Body (non-appendicular structures)

54. Metamerism of main body [Z54]: (0) absent; (1) present.
55. Number of body metameres [Z55]: (0) ≤ 20; (1) 21–24; (2) 25–37; (3) 38–43; (4) ≥ 44.
56. Number of dorsal divisions or tergites [Z56]: (0) ≤ 7; (1) 8–13; (2) 14–19; (3) 20–33; (4) ≥ 34.
57. Size change of main body along body axis [Z57]: (0) cylindrical; (1) broad anteriorly and tapering posteriorly.

Head (non-appendicular structures)

58. Anterior paired projections [Z58]: (0) absent; (1) present.
59. Head sclerotization [Z59]: (0) absent; (1) present.
60. Unsclerotized head part possessing non-appendicular proboscis with a clear posterior boundary [Z60]: (0) absent; (1) present.
61. Unsclerotized head part, anteriormost part adjoined to bases of frontalmost appendages [Z61]: (0) absent; (1) present.
62. Unsclerotized head part protruded [Z62]: (0) absent; (1) present.
63. Unsclerotized head part inflated, narrowed at base of protrusion [Z63]: (0) absent;

(1) present.

64. Unsclerotized head part elongated and tubular [Z64]: (0) absent; (1) present.
65. Anteriormost sclerite associated with eyes (ocular sclerites) [Z65]: (0) absent; (1) present.
66. Anteriormost sclerites forming a sclerite complex [Z66]: (0) absent; (1) present.
67. Configuration of anteriormost sclerites [Z67]: (0) central element oval, lateral elements connected by peduncular structures; (1) central ‘H-element’ and paired lateral ‘P-elements’.
68. Ocular sclerite as a dorsal plate with pronounced marginal rim covering anteriormost head part [Z68]: (0) absent; (1) present.
69. Expanded head sclerite with posterior notches accommodating eyes, covering a large head region [Z69]: (0) absent; (1) present.
70. Ocular sclerite accommodated by fused head shield [Z70]: (0) absent; (1) present.
71. Ocular sclerite accommodated by bivalved head carapace [Z71]: (0) absent; (1) present.
72. Ocular sclerite accommodated by semi-circular head carapace [Z72]: (0) absent; (1) present.
73. Ocular sclerite accommodated by notched fused head shield [Z73]: (0) absent; (1) present.
74. Ocular sclerite covered by true head shield [Z74]: (0) absent; (1) present.
75. Post-ocular tergal sclerotization in head [Z75]: (0) absent; (1) present.
76. Carapace connected to the head region at its anterior part, posterior part free [Z76]: (0) absent; (1) present.
77. Simple head carapace covering only a few anterior trunk tergites [Z77]: (0) absent; (1) present.
78. Simple head carapace, morphology [Z78]: (0) semi-circular; (1) heart-shaped.
79. Bivalved carapace [Z79]: (0) absent; (1) present.
80. Body length covered by bivalved carapace, length of anterior appendages excluded [Z80]: (0) > 70%; (1) 45–70%; (2) < 45%.
81. Head carapace or shield, straight medial hinge [Z81]: (0) absent; (1) present.

82. Valve shape of bivalved carapace [Z82]: (0) symmetrical respective to sagittal axis, ventral margins tight (‘*Isoxys* type’); (1) asymmetrical respective to sagittal axis, anteroventral margin tight, posteroventral margin ample (‘*Canadaspis* type’); (2) symmetrical respective to sagittal axis, ventral margins ample
83. Bivalved carapace, relative length of anterior spine compared to valve
84. Transverse ridges or notches as segmental impression on head shield [Z93]: (0) absent; (1) present. length [Z83]: (0) absent or tiny spines; (1) short spine; (2) long spine.
85. Bivalved carapace, relative length of posterior spine compared to valve length [Z84]: (0) absent or tiny spines; (1) short spine; (2) long spine.
86. Bivalved carapace, average length of valves in adults, anterior and posterior spines excluded [Z85]: (0) shorter, < 3.5 cm; (1) longer, > 4 cm.
87. Bivalved carapace covering cephalothorax only [Z86]: (0) absent; (1) present.
88. Bivalved carapace, posterior margin notched from dorsal view [Z87]: (0) absent; (1) present.
89. Isoxyid-type bivalved carapace, curvature of anterior dorsal margin of valve [Z88]: (0) almost straight; (1) curved.
90. Isoxyid-type bivalved carapace, relative lengths of anterior and posterior spines [Z89]: (0) posterior spine longer; (1) anterior spine longer.
91. Soft-tissue bundle into the anterodorsal end of bivalved carapace [Z90]: (0) absent; (1) present.
92. Fused head shield [Z91]: (0) absent; (1) present.
93. Head shield articulated with reduced anterior trunk tergites [Z92]: (0) absent; (1) present.
94.

    Remark. Present in several megacheirans and *Urokodia*
95. Glabellar furrows or lobes [Z94]: (0) absent; (1) present.
96. Anterior margin of head shield or carapace [Z95]: (0) convex; (1) almost straight; (2) subtriangular, rostral; (3) concave.
97. Additional anterior marginal structures of head shield [Z96]: (0) absent; (1)

pointed medially; (2) notched on single tergite.

98. Genal angles of head shield [Z97]: (0) rounded genal angles; (1) acute genal angles; (2) genal spines; (3) spine-like extension.

99. Medial notch on posterior margin of head shield [Z98]: (0) absent; (1) present.

100. Doublure of head shield [Z99]: (0) absent or weak; (1) strong.

101. Doublure of head carapace [Z100]: (0) absent or weak; (1) strong.

102. Structure of protocerebrum [Z101]: (0) cycloneuralian brain or non-dorsally restricted ganglionic protocerebrum; (1) dorsal restriction of protocerebrum.

103. Suboesophageal ganglion completely fused to thoracic plus abdominal ganglia [Z102]: (0) absent; (1) present.

104. Stomodeum extends to rostral margin of protocerebrum [Z103]: (0) absent; (1) present.

105. Number of segments in head region or covered by head shield or carapace [Z104]: (0) 1–2; (1) 3-4; (2) four and one-half; (3) 5; (4) ≥ 6.

Trunk or thorax (non-appendicular structures)

106. Metameres longest in middle trunk, shortening towards anterior and posterior [Z105]: (0) absent; (1) present.

107. Number and form of annuli between legs per metamere on trunk [Z106]: (0) > 7, narrow; (1) < 6, wide.

108. Dorsal/tergal boundary between head and thorax/trunk [Z107]: (0) absent; (1) present.

109. Articulation between head and thorax/trunk non-functional [Z108]: (0) absent; (1) present.

110. Occipital lobe [Z109]: (0) absent; (1) present.

111. Trunk narrowed anteriorly relative to head shield [Z110]: (0) absent; (1) present.

112. Dorsal/tergal boundary between thorax/trunk and pygidium/tail [Z111]: (0) absent; (1) present.

113. Dorsal/tergal boundaries in thorax/trunk [Z112]: (0) absent; (1) present.

114. Articulations of trunk tergites[Z113]: (0) tergites non-overlapping; (1) extensive overlap of tergites; (2) edge-to-edge pleural articulations.

115.Trunk tergites weakly sclerotized [Z114]: (0) absent; (1) present.

116.Articulating half rings on trunk segments [Z115]: (0) absent; (1) present.

117.Thickened inter-segmental rims on both trunk segments and head shield [Z116]: (0) absent; (1) present.

118.Straight cuticular ridge along with articulation between adjacent trunk segments [Z117]: (0) absent; (1) present.

119.Dorsal trunk effacement [S45]: (0) trunk with defined (separate or fused) tergite boundaries; (1) trunk tergite boundaries effaced laterally; (2) trunk tergite boundaries completely effaced.

Remark: For artiopodans.

120.Posterior tergal articulations functional, anterior ones variably fused [Z119]: (0) absent; (1) present.

121.Decoupling of multiple tergites and sternites/appendages in trunk [Z120]: (0) absent; (1) present.

122.Anterior tergal boundaries in trunk or thorax reflexed anterolaterally [Z121]: (0) absent, boundaries traverse or reflexed posterolaterally; (1) present.

123.Posterior tergites strongly curved compared to anterior tergites [Z122]: (0) absent; (1) present.

Remark: For leanchoilids and Haikoucaris of megacheirans, as well as some artiopodans.

124.Orientation of pleurae [Z123]: (0) horizontal; (1) around body.

125.Constricted trunk pleural region [Z124]: (0) absent; (1) present.

126.Size of pleurae [Z125]: (0) short, equal or inferior to body diameter; (1) long, exceeding body diameter.

127.Trunk tergite, shape [Z126]: (0) rounded; (1) pleural tips; (2) extended, forming spines.

128.Width change of trunk or thoracic tergites along the length [Z127]: (0) tapering or narrowing dramatically; (1) narrowing gradually, subequal widths.

129.Tergo-pleural rings in trunk [Z128]: (0) absent; (1) present.

130.Posterior trunk tergo-pleural rings strongly compacted, disc-like [Z129]: (0)

absent (1) present.

131. Raised axial region [S54]: (0) absent or weakly defined; (1) present and well defined.

132. Axial furrows [Z131]: (0) absent; (1) present.

133. Axial spine on non-terminal trunk tergite [Z132]: (0) absent; (1) present.

134. Number of trunk divisions or tergites [Z133]: (0) 0–1; (1) 2–4; (2) 5–11; (3) 12–19; (4) 20–41; (5) ≥ 42.

135. Number of prothoracic segments [Z134]: (0) 3; (1) 5 or 6.

Posterior body

136. Posterior trunk extension [Z135]: (0) absent; (1) present.

137. Abdomen as posterior differentiated segments [Z136]: (0) absent; (1) present.

138. Abdominal tergites narrowed in widths, distinguishing from thoracic tergites [Z137]: (0) absent; (1) present.

139. Number of limbless posterior tergites [Z138]: (0) 0–2; (1) 3–5; (2) ≥ 6.

140. Telson [Z139]: (0) absent; (1) present.

141. Telson fringed with setae [Z140]: (0) absent; (1) present.

142. Posterior tagmata with elongate lateral processes [Z141]: (0) absent; (1) present.

143. Posterior tagmata composed of three paired tail flaps [Z142]: (0) absent; (1) present.

144. Posteriormost lateral processes, morphology [Z143]: (0) absent; (1) present, isolated; (2) present, fused with middle projection.

145. Posteriormost lateral processes fused [Z144]: (0) absent; (1) present.

146. Posteriormost lateral processes recurved [Z145]: (0) absent; (1) present.

147. Posteriormost lateral processes with lanceolate tips [Z146]: (0) absent; (1) present.

148. Medial telson process [Z147]: (0) absent; (1) present.

149. Telson shape [Z148]: (0) paddle-shaped or lanceolate; (1) rod-like; (2) flap-shaped, medial and paired lateral processes fused; (3) paired lateral processes unfused or incompletely fused.

150. Posteriormost ovoid median plate attached to telson [Z149]: (0) absent: (1)

present.

151.Posteriormost tagma modified into a fluke [Z150]: (0) absent; (1) present.

152.Multiple posterior segments fused, forming a pygidium [Z151]: (0) absent; (1) present.

Remark: Here, pygidium is the posterior body part or shield and does not apply only to trilobites.

153.Pygidium with median keel [S64]: (0) absent; (1) present.

154.Pygidium with median broad-based spine [Z154; S65]: (0) absent; (1) present.

155.pygidium with lateral spines [Z155; S66]: (0) absent; (1) present.

156.Tailspine [S67]: (0) absent; (1) present.

Remark: Variable among euarthropods, especially the in the *Kerygmachela*, leanchoilids, and some of artiopodans.

157.Length of tailspine [S69]: (0) shorter than half the length of the trunk; (1) longer than half the length of the trunk.

158.Marginal spines on tailspine [S70]: (0) absent; (1) present.

159.Tail flaps [Z157]: (0) absent; (1) present.

160.Number of tail flap pairs [Z158]: (0) single; (1) multiple.

161.Furcae [Z159]: (0) absent; (1) present.

Appendages (general)

162.Paired appendages [Z160]: (0) absent; (1) present.

163.Lobopodous appendages [Z161]: (0) absent; (1) present.

164.Sclerotized appendages [Z162]: (0) absent; (1) present.

165.Arthropodized appendages [Z163]: (0) absent; (1) present.

166.Sclerotized head appendages [Z164]: (0) absent; (1) present.

167.Arthropodized head appendages [Z165]: (0) absent; (1) present.

168.Sclerotized trunk appendages [Z166]: (0) absent; (1) present.

169.Arthropodized trunk appendages [Z167]: (0) absent; (1) present.

Frontalmost appendages

170.Frontalmost appendages, position on head [Z168]: (0) lateral; (1) dorsolateral; (2) ventral.

171. Frontalmost appendages, orientation [Z169]: (0) non-specific or lateral; (1) downward; (2) upward.
172. Frontalmost appendages, segmental identity [Z170]: (0) protocerebral; (1) deuterocerebral.
173. Frontalmost appendages, arthropodization [Z171]: (0) absent; (1) present.

    Remark: *Urokodia* bearing unarthropodization antennae.
174. Frontalmost appendages, annulation [Z172]: (0) absent; (1) present.

    Remark: Present in the lobopodous forms and *Urokodia*.
175. Frontalmost appendages, composition of distal annuli or articulated podomeres, appendage terminal excluded [Z173]: (0) homonomous; (1) heteronomous.
176. Frontalmost appendages, absolute number of distal articulated podomeres or annuli [Z174]: (0) smooth or $\geq$ 16; (1) 8–15; (2) 5–7; (3) 4; (4) 3; (5) $\leq$ 2.
177. Frontalmost appendages, proximal shaft region differentiated from distal articulated podomeres [Z175]: (0) absent; (1) present.
178. Relative number of podomeres or annuli in frontalmost appendages compared to that in trunk endopods or legs [Z176]: (0) close; (1) significantly more; (2) significantly less; (3) reduced.
179. Frontalmost appendages specialised, with a unique morphology compared to all other appendages [Z177]: (0) absent; (1) present.
180. Frontalmost appendages, antenniform [Z178]: (0) absent; (1) present.
181. Frontalmost appendages, endites well-developed, raptorial [Z179]: (0) absent; (1) present.
182. Frontalmost appendages, terminal structures [Z180]: (0) similar to other podomeres; (1) claw; (2) cuticular spines.
183. Frontalmost appendages, terminal claw bearing multiple cusps [Z181]: (0) absent; (1) present.
184. Frontalmost appendages (unarthropodized), located at the protruded ‘neck’ [Z182]: (0) absent; (1) present.
185. Frontalmost appendages (unarthropodized), antenniform [Z183]: (0) absent; (1) present.

186. Frontalmost appendages (unarthropodized), tentacle-like [Z184]: (0) absent; (1) present.
187. Frontalmost appendages (unarthropodized), unspecialised lobopod [Z185]: (0) absent; (1) present.
188. Frontalmost appendages (unarthropodized), specialised raptorial lobopod [Z186]: (0) absent; (1) present.
189. Frontalmost appendages (unarthropodized), non-terminal spinous outgrowths [Z187]: (0) absent or invisible; (1) present.
190. Frontalmost appendages (arthropodized), number of podomeres bearing well-expanded endites, terminal podomere excluded [Z188]: (0) 0 or 1; (1) 2 or 3; (2) 4–7; (3) ≥ 8.
191. Frontalmost appendages (arthropodized), shaft endite [Z189]: (0) absent; (1) present.
192. Frontalmost appendages (arthropodized), differentiated main spines or main protrusions of endites [Z190]: (0) absent; (1) present.
193. Frontalmost appendages (arthropodized), relative length of endite to podomere height [Z191]: (0) absent or shorter; (1) comparable; (2) much longer.
194. Frontalmost appendages (arthropodized), relative width of base of endite to podomere length [Z192]: (0) endite absent or narrowed; (1) comparable, along the entire podomere.
195. Frontalmost appendages (arthropodized), morphology of enditic spines [Z193]: (0) absent; (1) spiky; (2) elongated, blade-like; (3) elongated, finger-like; (4) needle-like.
196. Frontalmost appendages (arthropodized), enditic spines originated from distal portion of podomere, tapering distally [Z194]: (0) absent; (1) present.
197. Frontalmost appendages (arthropodized), endites broadened laterally, overlapping with adjacent ones [Z195]: (0) absent; (1) present.
198. Frontalmost appendages (arthropodized), endites, change of lengths along the appendage [Z196]: (0) decreasing gradually towards tip; (1) alternating (short-long-short etc.).

199. Frontalmost appendages (arthropodized), endite pincer-like [Z197]: (0) absent; (1) present.

200. Frontalmost appendages (arthropodized), endites bearing well-developed auxiliary spines [Z198]: (0) absent; (1) present.

201. Frontalmost appendages (arthropodized), endites, auxiliary spines distribution [Z199]: (0) both anterior and posterior; (1) anterior only.

202. Frontalmost appendages (arthropodized), chelate or sub-chelate endites on distal podomeres [Z200]: (0) absent; (1) present.

203. Frontalmost appendages (arthropodized), length of basal podomere(s) [Z201]: (0) short; (1) elongated.

204. Frontalmost appendages (arthropodized), oblique arthrodial membrane in shaft region [Z202]: (0) absent; (1) present.

205. Frontalmost appendages (arthropodized), proximal podomeres differentiated and peduncle-like [Z203]: (0) absent; (1) present.

206. Frontalmost appendages (arthropodized), peduncle podomere morphology [Z204]: (0) short; (1) elongated.

207. Frontalmost appendages (arthropodized) geniculate at the middle pivot, forming an elbowed articulation [Z205]: (0) absent; (1) present.

208. Frontalmost appendages (arthropodized), elongate terminal podomere [Z206]: (0) absent; (1) present.

209. Frontalmost appendages (arthropodized), flagella [Z207]: (0) absent; (1) present.

210. Frontalmost appendages (arthropodized), length of flagella [Z208]: (0) midlength of trunk; (1) end of body.

211. Frontalmost appendages (arthropodized), dorsal spines [Z209]: (0) absent; (1) present.

212. Frontalmost appendages (arthropodized), length of multi-podomerous antenniform types compared with length of bivalved head carapace [Z210]: (0) short; (1) long.

213. Frontalmost appendages (arthropodized), raptorial device made of three to four podomeres with elongate endites [Z211]: (0) absent; (1) present.

214.Frontalmost appendages (arthropodized), chelate endites only on terminal podomeres, cheliceralike or chelicerae [Z212]: (0) absent; (1) present.

Post-oral appendages

215.Anteriormost appendages differentiated from posterior ones [Z23]: (0) absent; (1) present.

216.Differentiated multiple pairs of anterior homonomous unarthropodized appendages [Z214]: (0) absent; (1) present.

217.Differentiated multiple pairs of anterior homonomous unarthropodized appendages, number of pairs [Z215]: (0) two to three; (1) five to six.

218.Differentiated multiple pairs of anterior homonomous unarthropodized appendages, bearing spine-like long claws [Z216]: (0) absent; (1) present.

219.Multiple post-oral homonomous appendages (legs, flaps) accommodated to head region, differentiated from the frontalmost and other post-oral appendages [Z217]: (0) absent; (1) present.

220.Anterior homonomous appendages in head smaller, differentiated in size from posterior ones [Z218]: (0) absent; (1) present.

221.Anterior reduction of segments and/or appendages [Z219]: (0) absent; (1) present.

222.Second antennae on third head segment [Z220]: (0) absent; (1) present.

223.Specialised post-antennal appendages (SPAs) [Z221]: (0) absent; (1) present.

224.Morphology of appendages on third head segment [Z222]: (0) homonomous to posterior appendages; (1) heteronomous to posterior appendages, or reduced, forming intercalary segment.

225.Mandibles or paired mandible-like appendages [Z223]: (0) absent; (1) present.

226.Mandible morphology [Z224]: (0) basipodite with elaboration of proximal endite; (1) coxal endite embedded between the labrum and hypopharynx to form a chewing chamber.

227.Mandibular palp [Z225]: (0) absent; (1) present.

228.Specialised raptorial post-tritocerebral appendages [Z226]: (0) absent; (1) present.

229.Number of unique morphological types of anteriormost appendage pairs [Z227]: (0) 0; (1) 1; (2) 2; (3) ≥ 3.

230.Differentiation of trunk appendages in sets, each consisting of multiple appendages in similar morphology [Z228]: (0) absent; (1) present.

231.External metameric boundaries on unarthropodized trunk appendages [Z229]: (0) absent; (1) present.

232.Well-developed papillae or cuticular outgrowth on unsclerotized appendages [Z230]: (0) absent; (1) present.

233.Post-oral appendages, length [Z231]: (0) subequal along length of body; (1) anterior appendages about twice as long as posterior appendages.

234.Post-oral telescopic legs or lobopods [Z232]: (0) absent; (1) present.

235.Post-oral telescopic legs or lobopods, multiple rows of cuticular spines in feather-like arrangement [Z233]: (0) absent; (1) present.

236.Post-oral telescopic legs or lobopods bearing long setiform spines [Z234]: (0) absent; (1) present.

237.Post-oral telescopic legs or lobopods, relative widths [Z235]: (0) very narrowed; (1) narrower than trunk diameter; (2) comparable to trunk diameter.

238.Post-oral telescopic legs or lobopods, relative length [Z236]: (0) shorter or close to trunk diameter; (1) at least 1.5 times longer than trunk diameter; (2) state 0 or state 1.

239.Number of leg pairs on trunk terminal [Z237]: (0) one; (1) two.

240.Papillae or spines on legs [Z238]: (0) absent or tiny; (1) prominent.

241.Number of claw elements on leg or frontalmost appendage [Z239]: (0) none; (1) single; (2) double; (3) multiple.

242.Stacked structure of claws or sclerites [Z240]: (0) absent; (1) present.

243.Large distal claw elements with a wide base on appendages [Z241]: (0) absent; (1) present.

244.Number of claws on posteriormost lobopods [Z242]: (0) multiple; (1) single.

245.Posteriormost claws or legs orientated to the anterior [Z243]: (0) absent; (1) present.

246.Post-oral appendages, protopodite or gnathobase-like structures [Z244]: (0) absent; (1) present.

247.Sclerotization of protopodite or gnathobase-like structures [new]: (0) absent; (1) present.

Remark: Non-scleratization protopodite presented in *Urokodia*.

248.Annuli of protopodite or gnathobase-like structures [new]: (0) absent; (1) present.

Remark: Multiple annuli protopodite presented in *Urokodia*.

249.Protopodite or gnathobase, fusion of multiple podomeres [Z245]: (0) superficial repetitive structures invisible; (1) repetitive homonomous enditic structures visible; (2) multiple segmented podomeres and boundaries visible.

250.Protopodite, proximal endite [Z246]: (0) absent; (1) present.

251.Protopodite, coxa [Z247]: (0) absent; (1) present.

252.Biramous appendages with endopodites and exopodites [Z248]: (0) absent; (1) present.

253.Post-oral appendages, jointed legs or endopodites [Z249]: (0) absent; (1) present.

254.Post-oral appendages, endopodite or leg, proximal-distal differentiation of podomeres or annuli towards tip [Z250]: (0) more or less homonomous, tapering continuously; (1) differentiated, discontinuous change in length-width ratio of podomeres.

255.Post-oral appendages, endopodite, endites [Z251]: (0) absent or well-reduced; (1) distal spines only; (2) serrated rows of spines; (3) well-extended endite with a cluster of multiple spines.

256.Post-oral appendages, endopodite, number of podomeres with distal claw included [Z252]: (0) $\geq$ 12; (1) 8–11; (2) 7; (3) $\leq$ 6.

257.Post-oral appendages, endopodite, chelate terminal podomere [Z253]: (0) absent; (1) present.

258.Post-oral appendages, endopodite and protopodite as a whole, maximum number of podomeres [Z254]: (0) $\geq$ 13; (1) 9–12; (2) 8; (3) $\leq$ 7.

259.Protopodite and endopodite podomeres close in lengths [Z255]: (0) absent; (1) present.

260.Lateral flaps or exopodites [Z256]: (0) absent; (1) present.

261.Lateral flaps or exopodites, attachment [Z257]: (0) body flap; (1) protopodite

only.

262.Exopodite morphology, lateral flaps [Z258]: (0) absent; (1) present.

263.Exopodite morphology, lobe shape [Z259]: (0) lobe absent; (1) undivided lobe; (2) shorter proximal lobe and longer distal lobe; (3) proximal lobe no shorter than distal lobe.

264.Exopodite morphology, segmented podomeres [Z260]: (0) absent; (1) present.

265.Exopodite morphology, numerous annuli [Z261]: (0) absent; (1) present.

266.Multiple exopodite lobes or book gills [Z262]: (0) absent; (1) present.

267.Exopodite differentiated into proximal lobe bearing imbricated lamellar setae and distal lobe fringed by non-lamellar setae [Z263]: (0) absent; (1) present.

268.Proximal lobe of exopodite [Z264]: (0) flattened lobe; (1) narrowed lobe or shaft.

269.Distal lobe of exopodite [Z265]: (0) large; (1) small to moderate size.

270.Exopodites, anterior and posterior parts differentiation [Z266]: (0) absent; (1) present.

271.Exopodites, rays in a parallel row [Z267]: (0) absent; (1) present.

272.Exopodites, septa or narrowed proximal part [Z268]: (0) absent; (1) present.

273.Differentiation of setae along exopodite [Z269]: (0) absent; (1) present.

274.Trunk exopodite, setae [Z270]: (0) absent or short fine setae; (1) present.

275.Trunk exopodite, slightly separated oblanceolate setae [Z271]: (0) absent; (1) present.

276.Trunk exopodite, lamellar setae [Z272]: (0) absent; (1) present.

277.Trunk exopodite, widely spaced filamentous setae [Z273]: (0) absent; (1) present.

278.Non-lamellar marginal setae on exopodite [Z274]: (0) absent; (1) present.

279.Setal structures [Z275]: (0) absent; (1) present.

280.Setal structures, distribution [Z276]: (0) confined laterally, associated with lateral flaps or exopodites; (1) present dorsally.

281.Setal structures, fusion [Z277]: (0) unfused; (1) fused across the back.

282.Imbrication of setal structures [Z278]: (0) absent; (1) present.

283.Epipodite [Z279]: (0) absent; (1) present.

284.A series of long internal soft structures deep into trunk appendages [Z280]: (0)

absent; (1) present.

285.Lengths of appendages subequal along middle trunk, shortening towards anterior and posterior [Z281]: (0) absent; (1) present.

286.Appendages behind the third pair of appendages, morphology [Z282]: (0) homonomous, undifferentiated; (1) heteronomous, differentiated into various types.

287.Number of appendage pairs [Z283]: (0) ≤ 8; (1) 9–10; (2) 11–14; (3) 15 or 16; (4) ≥ 17.

Supplementary references

[S1] Hou, X., Chen, J, and Lu, H. (1989). Early Cambrian new arthropods from Chengjiang, Yunnan. Acta Palaeontol. 28, 42–57. 10.19800/j.cnki.aps.1989.01.004.

[S2] Zhang, X.L., Han, J., and Shu, D.G. (2002). New occurrence of the Burgess Shale arthropod *Sidneyia* in the Early Cambrian Chengjiang Lagerstätte (South China), and revision of the arthropod *Urokodia*. Alcheringa 26, 1–8. 10.1080/03115510208619239.

[S3] Berks, H.O., Nielsen M. L., Flannery Sutherland J., Nielsen A.T., Park Tae Yoon S., Vinther J. (2023). A possibly deep branching artiopodan arthropod from the A possibly deep branching artiopodan arthropod from the lower Cambrian Sirius Passet Lagerstätte (North Greenland). Pap. palaeontol. **9** (3). http://dx.doi.org/10.1002/spp2.1495.

[S4] Aria, C., and Caron, J.B. (2019). A middle Cambrian arthropod with chelicerae and proto-book gills. Nature 573, 586–589. https://www.nature.com/articles/s41586-019-1525-4.

[S5] Fortey, R.A. (1974). A new pelagic trilobite from the Ordovician of Spitsbergen, Ireland and Utah. Palaeontology 17, 111–124.

[S6] Salter, J.W. (1853). Figures and Descriptions Illustrative of British Organic Remains. (London, Printed for H. M. Stationery Off).

[S7] Zeng, H., Zhao, F.C., Niu, K.C., Zhu, M.Y., and Huang, D.Y. (2020). An early Cambrian euarthropod with radiodont-like raptorial appendages. Nature 588 (7836), 1–5. https://www.nature.com/articles/s41586-020-2883-7.

[S8] Schmidt, M., Hou, X.G., Zhai, D.Y., Mai, H.J., Belojevic, J., Chen, X.H., Melzer, R.R., Ortega-Hernández, J., and Liu, Y. (2021). Before trilobite legs: Pygmaclypeatus daziensis reconsidered and the ancestral appendicular organization of Cambrian artiopods. Phil. Trans. R. Soc. B 377, 20210030. http://dx.doi.org/10.1101/2021.08.18.456779.
